# Bimodal regulation of the PRC2 complex by USP7 underlies tumorigenesis

**DOI:** 10.1093/nar/gkab209

**Published:** 2021-04-13

**Authors:** Dongxue Su, Wenjuan Wang, Yongqiang Hou, Liyong Wang, Xianfu Yi, Cheng Cao, Yuejiao Wang, Huan Gao, Yue Wang, Chao Yang, Beibei Liu, Xing Chen, Xiaodi Wu, Jiajing Wu, Dong Yan, Shuqi Wei, Lulu Han, Shumeng Liu, Qian Wang, Lei Shi, Lin Shan

**Affiliations:** Department of Biochemistry and Molecular Biology, School of Basic Medical Sciences, Capital Medical University, Beijing 100069, China; State Key Laboratory of Cellular Stress Biology, Innovation Center for Cell Signaling Network, School of Life Sciences, Xiamen University, Xiamen, Fujian 361102, China; Department of Biochemistry and Molecular Biology, School of Basic Medical Sciences, Capital Medical University, Beijing 100069, China; State Key Laboratory of Cellular Stress Biology, Innovation Center for Cell Signaling Network, School of Life Sciences, Xiamen University, Xiamen, Fujian 361102, China; Department of Biochemistry and Molecular Biology, School of Basic Medical Sciences, Tianjin Medical University, Tianjin 300070, China; Core Facilities for Molecular Biology, Capital Medical University, Beijing 100069, China; School of Biomedical Engineering, Tianjin Medical University, Tianjin 300070, China; Department of Biochemistry and Molecular Biology, School of Basic Medical Sciences, Tianjin Medical University, Tianjin 300070, China; Department of Biochemistry and Molecular Biology, School of Basic Medical Sciences, Tianjin Medical University, Tianjin 300070, China; State Key Laboratory of Cellular Stress Biology, Innovation Center for Cell Signaling Network, School of Life Sciences, Xiamen University, Xiamen, Fujian 361102, China; Department of Biochemistry and Molecular Biology, School of Basic Medical Sciences, Capital Medical University, Beijing 100069, China; Department of Biochemistry and Molecular Biology, School of Basic Medical Sciences, Capital Medical University, Beijing 100069, China; Department of Biochemistry and Molecular Biology, School of Basic Medical Sciences, Capital Medical University, Beijing 100069, China; Department of Biochemistry and Molecular Biology, School of Basic Medical Sciences, Capital Medical University, Beijing 100069, China; Department of Biochemistry and Molecular Biology, School of Basic Medical Sciences, Capital Medical University, Beijing 100069, China; Department of Biochemistry and Molecular Biology, School of Basic Medical Sciences, Capital Medical University, Beijing 100069, China; Department of Biochemistry and Molecular Biology, School of Basic Medical Sciences, Capital Medical University, Beijing 100069, China; Department of Biochemistry and Molecular Biology, School of Basic Medical Sciences, Capital Medical University, Beijing 100069, China; Department of Biochemistry and Molecular Biology, School of Basic Medical Sciences, Capital Medical University, Beijing 100069, China; Department of Biochemistry and Molecular Biology, School of Basic Medical Sciences, Capital Medical University, Beijing 100069, China; Tianjin Medical University Cancer Institute and Hospital, Tianjin Key Laboratory of Cancer Prevention and Therapy, National Clinical Research Center for Cancer, Tianjin 300060, China; Department of Biochemistry and Molecular Biology, School of Basic Medical Sciences, Tianjin Medical University, Tianjin 300070, China; Department of Biochemistry and Molecular Biology, School of Basic Medical Sciences, Capital Medical University, Beijing 100069, China

## Abstract

Although overexpression of EZH2, a catalytic subunit of the polycomb repressive complex 2 (PRC2), is an eminent feature of various cancers, the regulation of its abundance and function remains insufficiently understood. We report here that the PRC2 complex is physically associated with ubiquitin-specific protease USP7 in cancer cells where USP7 acts to deubiquitinate and stabilize EZH2. Interestingly, we found that USP7-catalyzed H2BK120ub1 deubiquitination is a prerequisite for chromatin loading of PRC2 thus H3K27 trimethylation, and this process is not affected by H2AK119 ubiquitination catalyzed by PRC1. Genome-wide analysis of the transcriptional targets of the USP7/PRC2 complex identified a cohort of genes including *FOXO1* that are involved in cell growth and proliferation. We demonstrated that the USP7/PRC2 complex drives cancer cell proliferation and tumorigenesis *in vitro* and *in vivo*. We showed that the expression of both USP7 and EZH2 elevates during tumor progression, corresponding to a diminished FOXO1 expression, and the level of the expression of USP7 and EZH2 strongly correlates with histological grades and prognosis of tumor patients. These results reveal a dual role for USP7 in the regulation of the abundance and function of EZH2, supporting the pursuit of USP7 as a therapeutic target for cancer intervention.

## INTRODUCTION

Polycomb repressive complex 2 (PRC2), a multi-protein assembly composed of EZH2, EED, SUZ12, RbAp46 and RbAp48, is required for transcriptional repression of key regulators involved in animal development (1). As the catalytic core, EZH2 in PRC2 acts to methylate H3K27 and functions to facilitate chromatin compaction and gene silencing (2). In the past years, extensive studies have implicated EZH2 in the development and progression of various malignancies including prostate cancer (3), breast cancer (4), endometrial cancer (5) and melanoma (6). Elevated expression of EZH2 is a distinct feature of these cancer types and is often correlated with a poor prognosis (7,8). Thus, understanding the regulation of the abundance and activity of EZH2 is of great importance to not only the delineation of the pathophysiological function of EZH2 but also the prevention and treatment of various cancers.

Ubiquitination levels are balanced by ubiquitinating enzymes, including E1, E2 and E3 (9), and deubiquitinating enzymes (DUBs) (10). Among known DUBs, ubiquitin-specific protease 7 (USP7), also known as herpes virus-associated ubiquitin-specific protease (HAUSP) (11), as it was originally identified as a herpes simplex virus type 1 Vmw110-interacting protein (12) and as a key regulator of p53 (13–15) is implicated in multiple cellular processes such as DNA repair (16), cytokinesis (17), DNA replication (18), innate immune responses (19), viral replication and infection (20) and mitosis progression (21). Meanwhile, it is demonstrated that USP7 formed a protein complex with guanosine 5′-monophosphate synthetase to catalyze the removal of H2BK120ub1 (22–24). It is also found that USP7 cooperates with EPOP and Elongin BC to affect H2B ubiquitination at promoters, which could indirectly affect H3K4me3 and RNA polymerase II-mediated transcription (25). In addition, reports also implicate that both catalytic activity and physical interaction of USP7 are necessary for UHRF1 ubiquitylation and stability regulation, which may impact cell proliferation (26). Recently, the elevated *USP7* expression was reported as a distinct gene signature of malignant melanoma (27). However, the specific contribution of USP7 to tumor progression is largely unexplored.

Ubiquitination also occurs on histones, especially H2A and H2B (28). Mono-ubiquitination of H2A and H2B impacts a wide range of chromatin-associated events such as transcription, chromatin organization and DNA replication (29). Dysregulation of H2A or H2B ubiquitination is associated with several pathological processes, particularly cancer (30,31). Analogously, the state of histone ubiquitination is balanced by the activities of the specific ubiquitin ligases and deubiquitinases. It seems that H2BK120ub1 and H2AK119ub1 are mutually exclusive, as these two histone marks are linked to transcriptional activation and repression, respectively. On the other hand, it has been reported that some specific recruiting factors are responsible for targeting PRC2 to genomic loci, then, PRC2 decorates lysine 27 of histone H3 with a trimethyl group (H3K27me3). Next, CBX proteins bind to the H3K27me3 mark and recruit canonical PRC1 complex to chromatin, leading to the deposition of the H2AK119ub1 (32). In addition, the non-canonical PRC1 (ncPRC1) complex lacks the CBX proteins. They contain RYBP or YAF2 that interact directly with RING1A or RING1B instead, which can also function as an E3 ubiquitin ligase to monoubiquitinate H2AK119. Furthermore, another distinctive feature of mammalian ncPRC1 complexes is that they contain any of PCGF1/3/5/6, each of which associated with specific additional subunits (33). Interestingly, USP7 belongs to the PCGF1-containing ncPRC1.1 that includes KDM2B, BCOR, SKP1, RYBP as well as YAF2 (34,35). As H2AK119ub1 and H3K27me3 are believed to be functionally coordinated in transcriptional repression (36), it will be interesting to gain a mechanistic insight into the coordination between H2BK120ub1 and H3K27me3.

FOXO family factors play important roles in a variety of biological processes, including cell cycle (37), apoptosis (38), oxidative and stress response (39,40). Among them, FOXO1 is involved in a wide range of organismal functions and is considered as a tumor suppressor (41–43). Dysregulation of FOXO1 is associated with multiple cancers such as prostate cancer (44), breast cancer (45) and endometrial cancer (42). However, the mechanistic involvement of FOXO1 in tumorigenesis remains to be further investigated.

Acquired resistance is a major problem limiting the long-term effectiveness of targeted cancer therapeutics (46,47). For example, melanoma is the most aggressive form of skin cancer with an annually increasing incidence (48). Unlike localized melanoma, which can be surgically resected, metastatic melanoma with extranodal spreading is typically treated with systemic means such as targeted therapy. Vemurafenib, a BRAF inhibitor, has been shown to improve outcomes in the majority of melanoma patients harboring the highly prevalent BRAF V600E mutation (49). However, most patients treated with vemurafenib show disease progression within 6–8 months due to invariable drug resistance (50). Clearly, identification of novel therapeutic targets is of great significance.

In this study, we found that USP7 is associated with the PRC2 complex. We demonstrated that USP7 deubiquitinates and stabilizes EZH2. Moreover, we showed that USP7-promoted H2BK120ub1 removal is required for PRC2 recruitment thus H3K27 methylation, and the process is independent on H2AK119ub1. We showed that USP7 coordinates with the PRC2 complex to control the expression of genes including *FOXO1* to promote cell proliferation and tumorigenesis *in vitro* and *in vivo*. Our data indicate that USP7 could regulate PRC2 complex by two ways to promote tumorigenesis, supporting the pursuit of USP7 as a potential target for cancer therapy.

## MATERIALS AND METHODS

### Antibodies and reagents

The sources of antibodies against the following proteins were as follows: HA (sc-805) from Santa Cruz Biotechnology; PCGF1 (PA5-49390, for WB), β-actin (A1978), EZH2 (AV38470, for IHC), RYBP (PRS2227, for WB) and FLAG (F3165) from Sigma; USP7 (05-1946, for WB, IF and IP) and H2AK119ub1 (ABE569, for WB and ChIP) from Millipore; Ubiquityl-Histone H2B (K120) (#5546, for WB, IF and ChIP), SKP1 (#2156, for WB), Histone H2A (#2578, for WB and ChIP), RNF20 (#11974, for WB), RNF40 (#12187, for WB) and FOXO1 (#2880, for WB and IHC) from Cell Signaling Technology; KDM2B (ab234082, for WB), SUZ12 (ab175187, for WB and IP), H3K27me3 (ab6002, for WB, IF, ChIP, and ChIP-seq), H2B (ab64165, for WB and ChIP), DDDDK-tag (ab1257, for IF) and histone H3 (ab1791, for WB and ChIP) from Abcam; BCOR (12107-1-AP, for WB) from Proteintech; USP7 (A300-033A, for IP, IF, IHC and ChIP), RING1A (A303-552A, for WB) and RING1B (A302-869A, for WB) from Bethyl Lab; Myc (M047-3) from MBL; EED (GTX33168, for WB and IP) and YAF2 (GTX115355, for WB) from GeneTex; EZH2 (612667, for WB, IP and IF) from BD Transduction Laboratories; and EZH2 (39901, for ChIP and ChIP-seq) from Active Motif. Anti-HA affinity gel (E6779), anti-FLAG M2 affinity gel (A2220), 3 × FLAG peptide (F4799), MG132 (SML1135), blasticidin (15205), puromycin (P8833) and doxycycline (D9891) were purchased from Sigma. Ni-NTA Purification System (K950-01) was purchased from Invitrogen. CHX and HBX 41,108 were purchased from TOCRIS. GNE-6640 was purchased from Glixx Laboratories. Dabrafenib (GSK2118436) and vemurafenib were purchased from Selleck.

### Plasmids

The FLAG- or Myc-tagged USP7/WT was amplified from *USP7* cDNA kindly provided by Dr. Yang Shi (Harvard Medical School, Boston, MA, USA) and Dr. Ruaidhri J. Carmody (University of Glasgow, Scotland, UK) and integrated into pLVX-Tight-Puro, pLenti-hygro, or pcDNA3.1 vector, while the FLAG-tagged USP7/C223S carried by pLVX-Tight-Puro or pLenti-hygro vector was generated by a quick-change point mutation assay. His-tagged USP7/WT, USP7/C22 and deletion mutants of USP7 were carried by pFastBac-HTA vector. CRISPR/Cas9 constructs lentiCas9-Blast (Addgene plasmid # 52962) and lentiGuide-Puro (Addgene plasmid # 52963) were gifts from Dr. Feng Zhang (Broad Institute, Cambridge), and HA-tagged ubiquitin K48-only (Plasmid #17605) and K63-only (Plasmid #17606) were gifts from Dr. Ted Dawson (Johns Hopkins University School of Medicine, Baltimore, MD, USA). FLAG-tagged EZH2 carried by pLenti-hygro or pcDNA3.1 vector, GST-tagged deletion mutants of EZH2 in pGEX4T-3 vector, and FLAG-tagged SUZ12, EED, RNF20 or RNF40 in pcDNA3.1 vector were purchased from Youbio (Hunan, China).

### Cell culture

HEK293T, A375, PIG1 and Sf9 cells were obtained from American Type Culture Collection (Manassas, VA, USA) and cultured according to the manufacturer’s instructions. HaCAT cells were from the China Center for Type Culture Collection (CCTCC) and cultured according to the manufacturer’s instructions. Cell lines with Dox-induced protein expression were established in two steps. First, the cells were infected with a lentivirus carrying rtTA and subjected to neomycin selection. Subsequently, the established rtTA cells were infected with a virus carrying the pLenti-Tight-Puro vector encoding USP7/WT or USP7/C223S followed by puromycin selection. All of the cells integrated with rtTA were cultured in Tet Approved FBS and medium from Clontech. All the cells were authenticated by examination of morphology and growth characteristics and confirmed to be mycoplasma-free.

### *USP7* KO cell generation

*USP7* KO A375 cells were generated by co-transfection of the plasmid encoding FLAG-Cas9 (lentiCas9-Blast) and sgRNA plasmid (lentiGuide-Puro) targeting *USP7* (AATCAGATTCAGCATTGCAC). Forty-eight hours after transfection, the cells were selected by blasticidin (5 μg/ml) and puromycin (1 μg/ml) for 2 days.

### Immunopurification and silver staining

Lysates from A375 cells stably expressing FLAG-EZH2 were prepared by incubating the cells in lysis buffer containing protease inhibitor cocktail (Roche). Anti-FLAG immunoaffinity columns were prepared using anti-FLAG M2 affinity gel (Sigma) following the manufacturer’s protocols. Cell lysates were obtained from approximately 5 × 10^7^ cells and applied to an equilibrated FLAG column of a 1-ml bed volume to allow for adsorption of the protein complex to the column resin. After binding, the column was washed with cold PBS plus 0.2% Nonidet *P*-40. FLAG peptide (Sigma) was applied to the column to elute the FLAG protein complex according to the manufacturer’s instructions. The elutes were collected and visualized on a NuPAGE 4–12% Bis-Tris gel (Invitrogen) followed by silver staining with silver staining kit (Pierce). The distinct protein bands were retrieved and analyzed by LC-MS/MS.

### Nano-HPLC-MS/MS analysis

LC-MS/MS analysis was performed using a Thermo Finnigan LTQ linear ion trap mass spectrometer in line with a Thermo Finnigan Surveyor MS Pump Plus HPLC system. Generated tryptic peptides were loaded onto a trap column (300SB-C18, 5 × 0.3 mm, 5-μm particle size; Agilent Technologies, Santa Clara, CA, USA) connected through a zero-dead-volume union to the self-packed analytical column (C18, 100 μm i.d × 100 mm, 3-μm particle size; SunChrom, Germany). The peptides were then eluted over a gradient (0–45% B in 55 min, 45–100% B in 10 min, where B = 80% acetonitrile, 0.1% formic acid) at a flow rate of 500 nl/min and introduced online into the linear ion trap mass spectrometer (Thermo Fisher Corporation, San Jose, CA, USA) using nano electrospray ionization. Data-dependent scanning was incorporated to select the five most abundant ions (one microscan per spectrum; precursor isolation width, 1.0 *m*/*z*; 35% collision energy, 30 ms ion activation; exclusion duration, 90 s; repeat count, one from a full-scan mass spectrum for fragmentation by collision-induced dissociation. MS data were analyzed using SEQUEST (v. 28) against the NCBI human protein database (downloaded 14 December 2011; 33 256 entries), and results were filtered, sorted and displayed using the Bioworks 3.2. Peptides (individual spectra) with Preliminary Score (Sp) ≥ 500; Rank of Sp (RSp) ≤ 5; and peptides with +1, +2 or +3 charge states were accepted if they were fully enzymatic and had a cross correlation (Xcorr) of 1.90, >2.75 and >3.50, respectively. At least two distinct peptides were assigned to each identified protein. The following residue modifications were allowed in the search: carbamidomethylation on cysteine as a fixed modification and oxidation on methionine as variable modification. Peptide sequences were searched using trypsin specificity and allowing for a maximum of two missed cleavages. Sequest was searched with a peptide tolerance of 3 Da and a fragment ion tolerance of 1.0 Da.

### FPLC chromatography

A375 nuclear extracts and FLAG-USP7-containing protein complexes were applied to a Superpose 6 size exclusion column (GE Healthcare) that had been equilibrated with dithiothreitol-containing buffer and calibrated with protein standards (Amersham Biosciences). The column was eluted at a flow rate of 0.5 ml/min and fractions were collected.

### Immunoprecipitation

Cell lysates were prepared by incubating the cells in NETN buffer (50 mM Tris-HCl, pH 8.0, 150 mM NaCl, 0.2% Nonidet *P*-40, 2 mM EDTA) in the presence of protease inhibitor cocktails (Roche) for 20 min at 4°C, followed by centrifugation at 14 000 × *g* for 15 min at 4°C. For IP, approximately 500 μg of protein was incubated with control or specific antibodies (1–2 μg) for 12 h at 4°C with constant rotation; 50 μl of 50% protein G magnetic beads (Invitrogen) was then added and the incubation was continued for an additional 2 h. The beads were washed five times using the lysis buffer. Between washes, the beads were collected by a magnetic stand (Invitrogen) at 4°C. The precipitated proteins were eluted from the beads by re-suspending the beads in 2 × SDS-PAGE loading buffer and boiling for 5 min. The boiled immune complexes were subjected to SDS-PAGE followed by IB with appropriate antibodies.

### *In vivo* deubiquitination assay

Cells with different treatments were lysed in RIPA buffer containing 50 mM Tris-HCl (pH 7.4), 150 mM NaCl, 1% NP-40, 0.1% SDS and protease inhibitor at 4°C for 30 min with rotation, and centrifuged at 20 000 × *g* for 15 min. Approximately 0.5–1.5 mg of cellular extracts were immunoprecipitated with anti-FLAG agarose affinity gel for 2 h. The beads were then washed five times with RIPA buffer, boiled in SDS loading buffer and subjected to SDS-PAGE followed by IB.

### *In vitro* deubiquitination assay

HA-Ub–conjugated FLAG-EZH2 was purified from A375 cells in high-salt and detergent buffer, and His-tagged USP7/WT or USP7/C223S was affinity purified using nickelchelating resin from extracts of baculovirus-infected insect cells. Recombinant EZH2-Ub and USP7/WT or USP7/C223S were then incubated in DUB buffer (50 mM Tris-HCl, pH 8.0; 50 mM NaCl; 1 mM EDTA; 10 mM DTT and 5% glycerol) at 37°C for 2 h. The reactions were stopped by boiling for 5 min in 5 × SDS-PAGE loading buffer, and the boiled protein complexes were subjected to SDS-PAGE followed by IB with appropriate antibodies.

### Recombinant protein purification and pull-down assays

Recombinant baculovirus carrying full-length USP7/WT or deletion mutants of *USP7* were generated with the Bac-to-Bac System (Invitrogen). Infected Sf9 cells were grown in spinner culture for 48–96 h at 27°C and His-tagged protein-purified using Ni^2+^-NTA agarose (Invitrogen) according to standard procedures. For the His pull-down assay, His-tagged protein was incubated with recombinant EZH2, SUZ12, or EED that was *in vitro-*transcribed and translated according to the manufacturer’s procedures (TNT T7 Quick Coupled Transcription/Translation Kit; Promega, Leiden, the Netherlands) at 4°C overnight. GST-fusion proteins were purified from *Escherichia coli* by glutathione-Sepharose 4B beads (GE Healthcare) and then washed with high salt buffer (20 mM Tris-HCl pH 7.4, 0.1 mM EDTA, and 300 mM NaCl). For the GST pull-down assay, GST-fusion proteins were incubated with *in vitro* transcribed and translated proteins at 4°C overnight. The beads were washed three times, then boiled in SDS loading buffer, and subjected to SDS-PAGE followed by immunoblotting.

### RNA interference

All siRNA transfections were performed using Lipofectamine RNAi MAX (Invitrogen) following the manufacturer’s recommendations. The final concentration of the siRNA molecules was 10 nM, and the cells were harvested 72–96 h later according to the experimental purpose. The USP7 siRNA was a mixture of individual siRNAs against USP7, and the EZH2 siRNA was a mixture of individual siRNAs against EZH2. The siRNA sequences are shown in Supplementary Table S3.

### Lentiviral production

The shRNAs targeting *USP7* (Sigma), *EZH2*, *FOXO1* or *p21* in pLKO vector or vectors encoding rtTA, USP7, EZH2 carried by pLenti vectors, as well as three assistant vectors (pMDLg/pRRE, pRSV-REV and pVSVG) were transiently transfected into HEK293T cells. Viral supernatants were collected 48 h later, clarified by filtration and concentrated by ultracentrifugation. The shRNA sequences are shown in Supplementary Table S4.

### qRT-PCR

Total cellular RNA was isolated with TRIzol reagent (Invitrogen) and used for first-strand cDNA synthesis with the Reverse Transcription System (Roche). Quantitation of all gene transcripts was performed by qPCR using a Power SYBR Green PCR Master Mix (Roche) and an ABI PRISM 7500 sequence detection system (Applied Biosystems) with the expression of *GAPDH* as the internal control. The qRT-PCR primers are shown in Supplementary Table S5.

### Chromatin immunoprecipitation

Approximately 10 million cells were cross-linked with 1% formaldehyde for 10 min at room temperature and quenched by the addition of glycine to a final concentration of 125 mM for 5 min. The fixed cells were resuspended in SDS lysis buffer (1% SDS, 5 mM EDTA, 50 mM Tris-HCl, pH 8.1) in the presence of protease inhibitors and subjected to 3 × 10 cycles (30 s on and 30 s off) of sonication (Bioruptor, Diagenode) to generate chromatin fragments of ∼300 bp in length. Lysates were diluted in buffer containing 1% Triton X-100, 2 mM EDTA, 20 mM Tris-HCl, pH 8.1, 150 mM NaCl. For IP, the diluted chromatin was incubated with control or specific antibodies (2 μg) for 12 h at 4°C with constant rotation; 50 μl of 50% protein G magnetic beads was then added and the incubation was continued for an additional 2 h. The beads were washed with the following buffers: TSE I (0.1% SDS, 1% Triton X-100, 2 mM EDTA, 20 mM Tris-HCl, pH 8.1, 150 mM NaCl), TSE II (0.1% SDS, 1% Triton X-100, 2 mM EDTA, 20 mM Tris-HCl, pH 8.1, 500 mM NaCl), buffer III (0.25 M LiCl, 1% NP-40, 1% sodium deoxycholate, 1 mM EDTA, 10 mM Tris-HCl, pH 8.1) and Tris-EDTA buffer. Between washes, the beads were collected by a magnetic stand at 4°C. The pulled-down chromatin complex together with the input were de-crosslinked at 70°C for 2 h in elution buffer (1% SDS, 5 mM EDTA, 20 mM Tris-HCl pH 8.1, 50 mM NaCl, 0.1 mg/ml Proteinase K). Eluted DNA was purified with a PCR purification kit (Qiagen) and analyzed by qPCR.

### ChIP sequencing, qChIP and ChIP/Re-ChIP

A375 cells or A375 cells expressed Control shRNA, USP7 shRNA and USP7 shRNA/FLAG-EZH2 were maintained in DMEM supplemented with 10% FBS. Approximately 5 × 10^7^ cells were used for each ChIP-Seq assay. The chromatin DNA was precipitated by polyclonal antibodies against USP7, EZH2, H3K27me3 or H2BK120ub1. The DNA was purified with a Qiagen PCR purification kit. In-depth whole-genome DNA sequencing was performed by BGI (Wuhan, China) and CapitalBio Corporation (Beijing, China). The raw sequencing image data were examined using the Illumina analysis pipeline with a *P*-value cutoff of 10^–3^, aligned to the unmasked human reference genome (UCSC GRCh37, hg19) using Bowtie2 and further analyzed by MACS2 (Model-based Analysis for ChIP-Seq, https://github.com/taoliu/MACS). Enriched binding peaks were generated after filtering through the input data. The genomic distribution of USP7- and EZH2-binding sites was analyzed by ChIPseeker, an R package for ChIP peak annotation, comparison and visualization (http://bioconductor.org/packages/release/bioc/html/ChIPseeker.html). DNA-binding motifs of USP7 and EZH2 were analyzed by MEME (http://meme-suite.org/tools/meme). For visualization of ChIP-Seq data, we generated a bigwig track heatmap for genome browsers using deepTools (version 2.2.0). Sequencing density of each ChIP-seq sample was calculated over every continuous 10-bp window across the genome and normalized by RPGC (reads per genome coverage) method. Density profiles across the USP7 specific binding peaks of ChIP-seq samples were extracted by manually stretching the peak summits to 3000-bp in both directions. Eluted DNA was purified using a PCR purification kit (QIAGEN), and qChIPs were performed using the TransStart Top Green qPCR Supermix (TransGen Biotech) by qPCR on the ABI 7500-FAST System. Re-ChIPs were performed essentially in the same manner as the primary IPs. Bead eluates from the first immunoprecipitation were incubated with 10 mM DTT at 37°C for 30 min and diluted 1:50 in dilution buffer (1% Triton X-100, 2 mM EDTA, 150 mM NaCl, 20 mM Tris-HCl, pH 8.1) followed by re-IP with the secondary antibodies. The final elution step was performed using 1% SDS solution in Tris-EDTA buffer, pH 8.0. The qChIP PCR primers are listed in Supplementary Table S6.

### Colony formation assay

A375 cells infected with the lentiviruses carrying the indicated shRNAs were treated with different doses of the chemotherapy drug dabrafenib or vemurafenib. The cells were then maintained for 14 days, fixed with methanol, and stained by crystal violet.

### Tissue specimens

The tissue samples were obtained from surgical specimens from patients with nevus, malignant melanoma and metastasis melanoma. The samples were frozen in liquid nitrogen immediately after surgical removal and maintained at −80°C until protein extraction. Human skin tissues were prepared; incubated with antibodies against USP7, EZH2 or FOXO1; and processed for IHC with standard DAB staining protocols. Images for adjacent normal (27), nevus (13), malignant melanoma (46) and metastasis melanoma (10) samples were collected under microscopy with 200× magnification. The image quality was evaluated and the background with uneven illumination was corrected with Image-Pro Plus software. The normal skin cells, nevus cells or melanoma cells were selected as the region of interest (ROI) according to the morphological features of the tissue or cells. The scores of the stained sections were determined by evaluating the mean intensity and nuclear staining extent of immunopositivity following the instructions of Image-Pro Plus software.

### Tumor xenografts

A375 cells were plated and infected *in vitro* with mock or lentiviruses carrying Control shRNA, USP7 shRNA or EZH2 shRNA together with FOXO1 shRNA. Forty-eight hours after infection, 3 × 10^6^ viable A375 cells in 100 μl PBS were injected into the 6–8-week-old female athymic mice (BALB/c; Charles River, Beijing, China). One week after inoculation, the mice were subjected to a feed vehicle or dabrafenib (30 mg/kg body weight) daily for half the mice in each group, and then tumor growth and body weight were monitored over the following 3 weeks. Five animals per group were used in each experiment. Tumors were measured weekly using a Vernier caliper and the volume was calculated according to the formula: π/6 × length × width^2^. The measurement and data processing were performed in a blinded manner to the group.

### Statistics

Data from biological triplicate experiments are presented with error bar as mean ± SD. Two-tailed unpaired Student’s *t*-test was used for comparing two groups of data. Analysis of variance (ANOVA) with Bonferroni’s correction was used to compare multiple groups of data. A *P* value of <0.05 was considered significant. All the statistical testing results were determined by SPSS software. Before statistical analysis, variation within each group of data and the assumptions of the tests were checked.

### Study approval

All animal handling and experiments were approved by the Animal Care Committee of Capital Medical University. The collection and analysis of human tissue samples were approved by the Ethics Committee of the Capital Medical University, and informed consent was obtained from all patients.

## RESULTS

### USP7 is physically associated with the PRC2 complex

**Figure 1.**
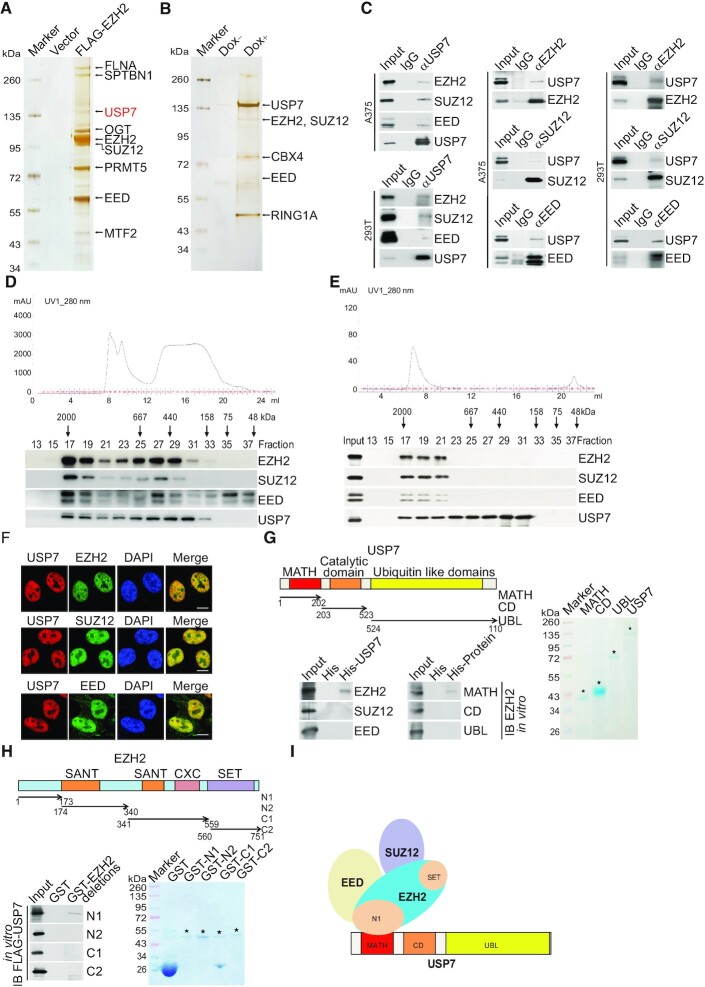
USP7 is physically associated with the PRC2 complex. (**A**) Immunopurification and mass spectrometry analysis of EZH2-containing protein complex. (**B**) Immunopurification and mass spectrometry analysis of USP7-containing protein complex. (**C**) Co-IP analysis of the association between USP7 and PRC2 complex. Whole cell lysates from A375 cells and HEK293T cells were immunoprecipitated (IP) then immunoblotted (IB) with antibodies against the indicated proteins. (**D**) Fast protein liquid chromatography analysis of the native protein complex. Chromatographic elution profiles (upper panel) and western blotting analysis (lower panel) of the chromatographic fractions with antibodies against the indicated proteins are shown. Equal volumes from each fraction were analyzed and the elution positions of calibration proteins with known molecular masses (kDa) are indicated. (**E**) Experiments analogous to (**D**) were performed with the USP7-containing protein complex purified from FLAG-USP7-expressing A375 cells. (**F**) Confocal microscopy analysis of USP7 and PRC2 complex subcellular localization. A375 cells were fixed and immunostained with antibodies against the indicated proteins; scale bar: 10 μm. (**G**) His-pull down assays with full-length or deletion mutants of USP7 purified from Sf9 insect cells and *in vitro*-transcribed/translated proteins as indicated. The asterisks indicate the recombinant proteins stained by Commassie Blue. (**H**) GST pull-down assays with bacterially expressed GST-fused deletion mutants of EZH2 and *in vitro-*transcribed/translated FLAG-USP7. The asterisks indicate the recombinant proteins stained by Commassie Blue. (**I**) Illustration of the molecular interfaces required for the association of USP7 with the PRC2 complex.

In an effort to better understand the biological function of EZH2 and to investigate its role in the development of cancer, we generated a human malignant melanoma A375 cell line stably expressing FLAG-EZH2. Whole-cell extracts from these cells were subjected to affinity purification using an anti-FLAG affinity gel. Mass spectrometric (MS) analysis showed that EZH2 was co-purified with SUZ12 and EED, the other subunits of the PRC2 complex (Figure 1A). Interestingly, the deubiquitinase USP7 was also identified in the EZH2-containing protein complex with high peptides coverage (Figure 1A). Additional proteins including OGT and SPTBN1 were also detected in the EZH2-containing complex (Figure 1A). To confirm the physical interaction of EZH2 with USP7, we generated an A375 cell line with doxycycline (Dox)-inducible expression of stably integrated FLAG-USP7. Similarly, the MS analysis of the whole-cell extracts from these cells with or without Dox-inducible expression of FLAG-USP7 showed that USP7 was co-purified with the PRC2 complex, as well as several other proteins (Figure 1B). The detailed result of the mass spectrometric analysis is provided in Supplementary Table S1.

To confirm the *in vivo* interaction of USP7 with the PRC2 complex, total proteins from A375 and 293T cells were extracted and co-immunoprecipitation (co-IP) was performed with antibodies detecting the endogenous proteins. Immunoprecipitation (IP) with antibodies against EZH2, SUZ12 or EED representing PRC2 followed by immunoblotting (IB) with antibodies against USP7 demonstrated that PRC2 core components were efficiently co-immunoprecipitated with USP7. Reciprocally, IP with antibodies against USP7 followed by IB with antibodies against EZH2, SUZ12 or EED confirmed that USP7 was efficiently co-immunoprecipitated with the PRC2 complex. These results suggest that the PRC2 complex interacts with USP7 *in vivo* (Figure 1C).

To further support the physical interaction of USP7 with the PRC2 complex, fast protein liquid chromatography (FPLC) was performed with A375 nuclear extracts using a Superpose 6 column and a high salt extraction and size-exclusion approach. Native USP7 in A375 cells was eluted with an apparent molecular mass much greater than that of the monomeric protein, and the chromatographic profile of USP7 largely overlapped with that of EZH2, SUZ12, and EED (Figure 1D). Furthermore, the majority of the purified FLAG-USP7 existed in a multiprotein complex, which peaked in fractions 17–19 with the PRC2 complex (Figure 1E). Consistently, immunofluorescent (IF) staining with antibodies against endogenous EZH2, SUZ12, EED or USP7 showed that the PRC2 complex and USP7 were colocalized in the nucleus of A375 cells (Figure 1F).

To substantiate the physical interaction of USP7 with the PRC2 complex *in vivo* and to understand the molecular detail involved in this interaction, *in vitro* pull-down experiments were carried out with full-length USP7 purified from the Sf9 insect cells and recombinant EZH2, SUZ12 or EED. The results showed that USP7 directly interacts with EZH2, but not with the other proteins tested (Figure 1G). Further pull-down experiments with the N-terminal meprin and TNF receptor-associated factor (TRAF) homology (MATH) domains, the catalytic domain (CD) and the C-terminal ubiquitin-like domain (UBL) of USP7 (51) showed that the MATH domain of USP7 is responsible for its interaction with EZH2 (Figure 1G). Moreover, glutathione S-transferase (GST) pull-down with GST-fused EZH2 N1 domain (aa 1-173), EZH2 N2 domain (aa 174–340), EZH2 C1 domain (aa 341–559), EZH2 C2 domain (aa 560–751) (52), and *in vitro*-transcribed/translated USP7 revealed that USP7 directly interacts with the N1 domain of EZH2 (Figure 1H). Together, these results not only support the specific interaction between USP7 and the PRC2 complex but also reveal the molecular interface involved in the formation of the USP7/PRC2 complex, as schematically summarized in Figure 1I.

### USP7 is functionally linked to the stabilization and deubiquitination of EZH2

**Figure 2.**
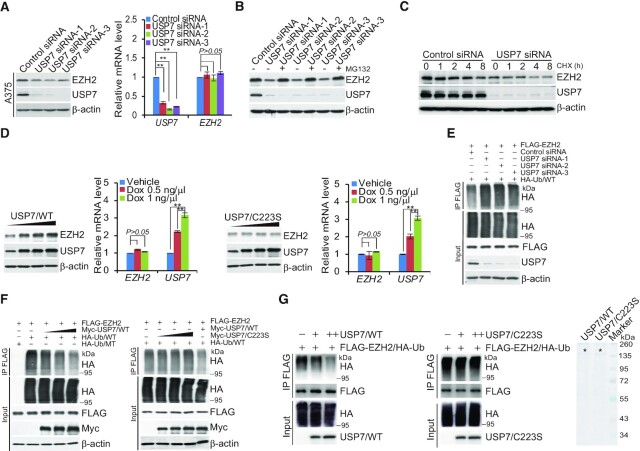
USP7 promotes deubiquitination and stabilization of EZH2. (**A**) A375 cells transfected with control siRNA or different sets of USP7 siRNAs were collected and analyzed by western blotting and quantitative reverse transcription (qRT)-PCR. Each bar represents the mean ± SD from biological triplicate experiments. **P < 0.01, by one-way ANOVA. (**B**) A375 cells transfected with Control siRNA or USP7 siRNAs were treated with DMSO or proteasome inhibitor MG132 (10 μM). Cellular extracts were prepared and analyzed by western blotting. (**C**) A375 cells were transfected with Control siRNA or USP7 siRNA followed by treatment with cycloheximide (CHX, 50 μg/ml), and harvested at the indicated time points followed by western blotting analysis. (**D**) A375 cells with Dox-inducible expression of FLAG-USP7/WT or FLAG-USP7/C223S cultured in the absence or presence of increasing amounts of Dox. Cellular extracts and total RNA were collected for western blotting and qRT-PCR analysis, respectively. Each bar represents the mean ± SD from biological triplicate experiments. **P < 0.01, one-way ANOVA. (**E**) A375 cells stably expressing FLAG-EZH2 co-transfected with Control siRNA or USP7 siRNAs and HA-Ub/WT. Cellular extracts were immunoprecipitated with anti-FLAG followed by immunoblotting with anti-HA. (**F**) A375 cells stably expressing FLAG-EZH2 were co-transfected with HA-Ub/WT or HA-Ub/MT and different amounts of Myc-USP7 or Myc-USP7/C223S as indicated. Cellular extracts were immunoprecipitated with anti-FLAG followed by immunoblotting with anti-HA. (**G**) *In vitro* deubiquitination assays were performed with HA-Ub-conjugated EZH2 purified from A375 cells using high-salt and detergent buffer and USP7/WT or USP7/C223S purified from baculovirus-infected insect cells. The asterisks indicate the recombinant proteins stained by Commassie Blue.

To investigate the functional significance of the physical interaction and spatial co-localization between USP7 and EZH2, we first examined the effect of USP7 on the expression of EZH2. Western blotting analysis of cellular lysates from A375 cells transfected with three independent sets of siRNAs targeting different regions of USP7 revealed that the level of EZH2 was significantly reduced upon USP7 depletion (Figure 2A), although quantitative reverse transcription PCR (qRT-PCR) indicates that USP7 knockdown did not alter *EZH2* mRNA levels in A375 cells (Figure 2A). Importantly, USP7 knockdown-associated reduction of EZH2 could be blocked by treatment of cells with the proteasome-specific inhibitor MG132 (Figure 2B), suggesting that the effect involves a proteasome-mediated protein degradation mechanism. Together, these observations indicate that the stability of EZH2 is regulated by USP7. To support this deduction, the potential of USP7 to modulate the steady-state level of EZH2 protein was assessed by cycloheximide (CHX) chase assays. After incubation with CHX, western blotting analysis revealed that USP7 depletion was clearly associated with a decreased half-life of EZH2 (Figure 2C). Moreover, it was evident that the expression levels of USP7 and EZH2 were correlated in multiple cell lines (Supplemental Figure S1A).

We next investigated whether USP7-promoted EZH2 stabilization is dependent on the enzymatic activity of USP7. To this end, we generated two stable A375 cell lines with Dox-inducible expression of wild type USP7 (USP7/WT) or a catalytically inactive mutant of USP7 (USP7/C223S), respectively. Western blotting showed a significant increase in the protein level of EZH2 in a Dox dose-dependent manner in cells expressing USP7/WT, but not USP7/C223S, whereas the mRNA level of *EZH2* was comparable in these two cell lines (Figure 2D). Moreover, the downregulation of EZH2 in USP7-deficient cells could be reversed by forced expression of USP7/WT, but not USP7/C223S (Supplementary Figure S1B). Furthermore, treatment of A375 cells with GNE-6640, a recently developed USP7 inhibitor (53), also resulted in a reduction of the protein level of EZH2 without affecting the *EZH2* mRNA level (Supplementary Figure S1C). Together, these results support a notion that USP7 regulates the stability of EZH2.

To gain mechanistic insight into USP7-regulated EZH2 stabilization, A375 cells stably expressing FLAG-EZH2 were co-transfected with USP7 siRNA and HA-tagged wild-type ubiquitin (Ub/WT). IP of the cellular lysates with anti-FLAG followed by IB with anti-HA showed that knockdown of USP7 led to an increased level of ubiquitinated EZH2 species (Figure 2E). Enzymatic inhibition of USP7 with GEN-6640 also resulted in a marked increase in the level of ubiquitinated EZH2 (Supplementary Figure S1D). In addition, experiments in FLAG-EZH2-expressing A375 cells co-transfected with different amounts of USP7 and HA-Ub/WT or an HA-tagged ubiquitin mutant (Ub/MT) with all lysine resides replaced by arginine showed that increased USP7 expression was associated with decreased levels of ubiquitinated EZH2 species (Figure 2F), whereas overexpression of USP7/C223S had no effects on the levels of ubiquitinated EZH2 (Figure 2F). Moreover, experiments in FLAG-EZH2-expressing A375 cells co-transfected with USP7 siRNAs and HA-tagged Ub/K48-only, a ubiquitin mutant with all lysine resides replaced by arginine except for residue 48 that signals targets proteins for degradation (54), or with HA-tagged Ub/K63-only, a ubiquitin mutant with all lysine resides replaced by arginine except for lysine 63, which is responsible for the proteasome-independent signals (54) showed that USP7 was capable of deubiquitinating K48-linked polyubiquitin chains but not K63-linked polyubiquitin chains (Supplementary Figure S1E). In addition, *in vitro* deubiquitination assays with HA-Ub-conjugated FLAG-EZH2 purified from A375 cells using high-salt and detergent buffer and His-USP7/WT or His-USP7/C223S purified from Sf9 cells were performed, the results revealed that USP7/WT was capable of deuibiquitinating EZH2, whereas USP7/C233S was not (Figure 2G). Analogously, we showed that SUZ12 and EED were not detected in HA-Ub-conjugated FLAG-EZH2 purified from A375 cells, suggesting that other PRC2 subunits were not pulled down in the FLAG IPs using high-salt and detergent buffer (Supplementary Figure S1F). Together, these results indicate that USP7 regulates the stability of EZH2 through its deubiquitinase activity.

### USP7-catalyzed H2BK120ub1 removal is a prerequisite for chromatin loading of PRC2 thus H3K27 trimethylation

**Figure 3.**
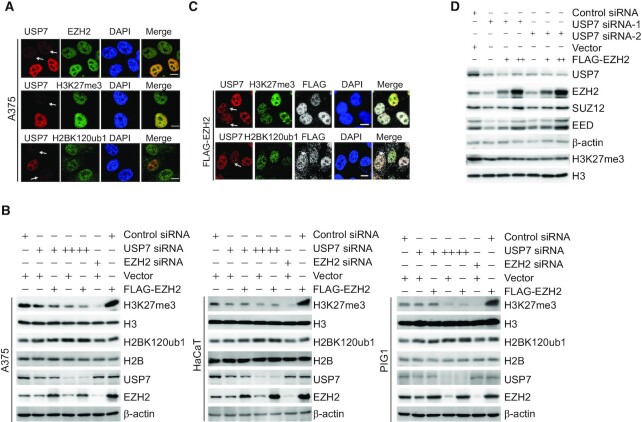
USP7 deubiquitinating H2BK120ub1 regulates the trimethylation of H3K27. (**A**) A375 cells transfected with USP7 siRNA were fixed and immunostained with the indicated antibodies followed by confocal microscopy analysis. The white arrows indicate cells with USP7 depletion to different extents; scale bar: 10 μm. (**B**) A375, HaCaT and PIG1 cells were co-transfected with Control siRNA, EZH2 siRNA, Vector or FLAG-EZH2 and different amounts of USP7 siRNA as indicated. Cellular extracts were collected and analyzed by western blotting. (**C**) A375 cells stably expressing FLAG-EZH2 transfected with USP7 siRNA, then fixed, and immunostained with the indicated antibodies followed by confocal microscopy analysis. The white arrows indicate cells with USP7 depletion to different extents; scale bar: 10 μm. (**D**) A375 cells were co-transfected with Control siRNA or different sets of USP7 siRNAs and different amounts of FLAG-EZH2 as indicated. Cellular extracts were collected and analyzed by western blotting.

Since the PRC2 complex is a well-characterized transcription regulatory assembly and USP7 is also implicated in transcription by virtue of its deubiquitinase activity toward H2BK120ub1 on chromatin (22–24), it will be interesting to know whether USP7 could influence the level of H3K27me3. To this end, USP7 was knocked down in A375 cells and immunofluorescent staining was performed with antibodies recognizing USP7, EZH2, H3K27me3 and H2BK120ub1. Confocal microscopy revealed that endogenous USP7 was colocalized with EZH2 in control cells, and in USP7-deficient A375 cells, the signals representing EZH2 and H3K27me3 significantly decreased, while H2BK120ub1 signals were elevated, as expected (Figure 3A and Supplementary Figure S2A), hinting a role for USP7 in the regulation of EZH2 recruitment and H3K27 trimethylation.

To support this, Control siRNA, EZH2 siRNA or USP7 siRNA with different dose were co-transfected with FLAG-Vector or FLAG-EZH2 in A375 cells. Western blotting showed that knockdown of USP7 resulted in a dose-dependent decreased level of H3K27me3, even when EZH2 was overexpressed (Figure 3B). Similar results were obtained in HaCaT cells, a spontaneously transformed aneuploid immortal keratinocyte cell line, and in human melanocyte PIG1 cells (Figure 3B). Immunofluorescent staining in A375 cells corroborated these observations (Figure 3C and Supplementary Figure S2B). Next, to further support the argument that USP7 plays a role in the regulation of H3K27 trimethylation dependent on stabilizing the EZH2, but not changing the integrity of PRC2 complex, USP7 was knocked down in A375 cells, and western blotting analysis showed that loss-of-function of USP7 was associated with a reduced expression of SUZ12 and EED, whereas EZH2 overexpression led to an increased expression of SUZ12 and EED, but not the level of H3K27me3 (Figure 3D). These results support the notion that USP7-promoted EZH2 stabilization is required for PRC2 complex formation and USP7 plays a key role in modulating H3K27me3 independent on the integral PRC2.

### Genome-wide identification of the transcriptional targets of the USP7/PRC2 complex

**Figure 4.**
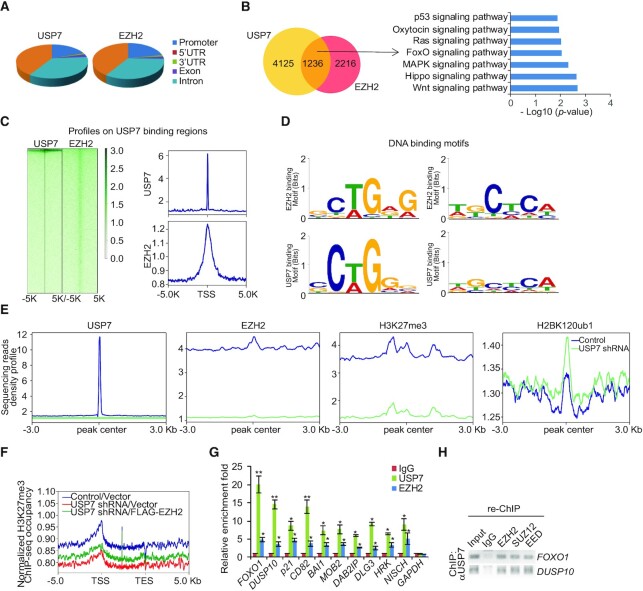
Genome-wide the transcription target analysis for the USP7/PRC2 complex. (**A**) Genomic distribution of USP7 and EZH2 determined by ChIP-seq analysis. (**B**) Venn diagram of overlapping promoters bound by USP7 and EZH2 in A375 cells. The numbers represent the number of promoters targeted by the indicated proteins. The clustering of the 1236 overlapping target genes of USP7/EZH2 into functional groups is shown. Detailed results of the ChIP-seq experiments are summarized in Supplementary Table S2. (**C**) ChIP-seq density heatmaps and profiles of EZH2 on USP7-binding sites. (**D**) The analysis of USP7-bound motifs and EZH2-bound motifs using MEME suite. (**E**) Visualization of those density profiles indicated the significant decrease of USP7, EZH2 and H3K27me3 densities and increase of H2BK120ub1 density around the USP7 specific peaks after knockdown of USP7. (**F**) Average occupancy of H3K27me3 of the USP7/EZH2-occupied genes in A375 cells expressed Control shRNA, USP7 shRNA or USP7 shRNA/FLAG-EZH2. (**G**) qChIP verification of the ChIP-seq results of the indicated genes with antibodies against the indicated proteins in A375 cells. Each bar represents the mean ± SD from biological triplicate experiments. *P < 0.05 and **P < 0.01, one-way ANOVA. (**H**) ChIP/Re-ChIP experiments on the promoters of the indicated genes with antibodies against the indicated proteins in A375 cells.

To explore the biological significance of the physical association of USP7 with the PRC2 complex, we next analyzed the genome-wide transcriptional targets of the USP7/PRC2 complex by chromatin immunoprecipitation (ChIP)-based deep sequencing (ChIP-seq). In these experiments, ChIP experiments were performed first in A375 cells with antibodies against USP7 and EZH2, respectively. Following ChIP, USP7- and EZH2-associated DNAs were amplified using non-biased conditions, labeled and then sequenced. We identified 19 189 USP7-specific binding peaks and 18 771 EZH2-specific binding sites (Figure 4A). The sequence reads from USP7 and EZH2 groups were then cross-analyzed for overlapping DNA sequences/gene promoters, and these promoters were considered to be the co-targets of the USP7/PRC2 complex. These experiments identified a total of 1236 distinct promoters that were targeted by the USP7/PRC2 complex (Figure 4B). The corresponding genes to these promoters were then classified into several cellular signaling pathways using the Gene Functional Annotation Tool available at the DAVID website with a *P*-value cut off of 10^−3^. These signaling pathways include Wnt, Hippo, MAPK, FoxO, Ras, oxytocin and p53 pathways that are known to be associated with tumorigenesis (Figure 4B). Importantly, genomic landscape analysis showed that EZH2 was significantly enriched in regions surrounding USP7 binding sites (Figure 4C). Analysis of the genomic binding signatures of USP7 and EZH2 revealed indeed similar binding motifs, such as GCTGAG or TGCTCA, for these two proteins (Figure 4D). Next, to further confirm that USP7 is required for the chromatin recruitment of EZH2, ChIP-seq experiments were performed in normal or USP7 knockdown A375 cells with specific antibodies against USP7, EZH2, H3K27me3 and H2BK120ub1. Consistent with our expectations, the analysis of ChIP-seq data showed that USP7 loss-of-function led to obviously reduction in USP7, EZH2 and H3K27me3 around the USP7-specific peaks, but an upregulation in H2BK120ub1 (Figure 4E). Importantly, even when EZH2 was overexpressed in USP7-deficient cells, the level of H3K27me3 on USP7/EZH2-occupied genes remained to be low (Figure 4F), further supporting USP7 is required for EZH2 loading and H3K27 trimethylation. Quantitative ChIP (qChIP) experiments on selected genes including *FOXO1, DUSP10, p21, CD82, BAI1, MOB2, DAB2IP, DLG3, HRK* and *NISCH* showed that USP7 and EZH2 indeed co-occupied the promoters of these genes, validating the ChIP-seq results (Figure 4G). To further support that USP7 and the PRC2 complex occupy the target genes in the context of the USP7-PRC2 complex, sequential ChIP or ChIP/Re-ChIP experiments were performed on the representative target genes *FOXO1* and *DUSP10*. In these experiments, soluble chromatins were first immunoprecipitated with antibodies against USP7, the immunoprecipitates were subsequently reimmunoprecipitated with appropriate antibodies. The results showed that, in precipitates, the *FOXO1* and *DUSP10* promoters that were immunorecipitated with antibodies against USP7 could be reimmunoprecipitated with antibodies against EZH2, SUZ12 and EED (Figure 4H), supporting the coexistence of USP7 and the PRC2 complex on the promoter of target genes and validating the finding that *FOXO1* and *DUSP10* are targeted by the USP7/PRC2 complex. Collectively, these results support the notion that USP7 and EZH2 physically interact and are functionally linked.

**Figure 5.**
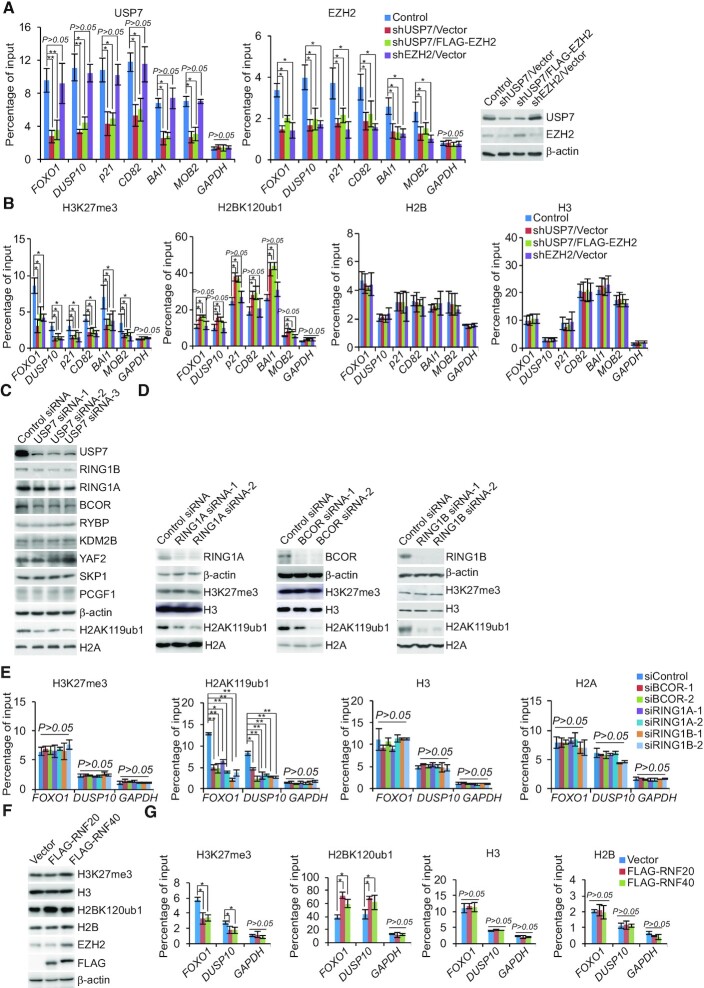
The assembly of the USP7/EZH2 complex on transcriptional targets. (**A** and**B**) qChIP analysis of selected promoters in the A375 cells after co-transfection with indicated shRNA and Vector or FLAG-EZH2 using the indicated antibodies. The knockdown efficiencies of USP7 and EZH2 were verified by western blotting. (**C**) Western blotting analysis of the expression of indicated proteins in A375 cells that transfected with different sets of USP7 siRNAs. (**D**) Western blotting analysis of the expression of H3K27me3 and H2AK119ub in A375 cells transfected with the indicated siRNAs. (**E**) A375 cells were transfected with indicated siRNAs for qChIP analysis on the selected promoters using antibodies against the indicated histone modification. (**F**) A375 cells transfected with FLAG-RNF20 or FLAG-RNF40 for the measurement of the indicated histone modification by western blotting. (**G**) A375 cells were transfected with the indicated expression vectors for qChIP analysis on the selected promoters using antibodies against the indicated histone modification. In A, B, E and G, data represent the mean ± SD from biological triplicate experiments. *P < 0.05 and **P < 0.01, one-way ANOVA.

Ubiquitination of histone H2B was reported to directly regulate H3K4 methylation to activate gene transcription (55,56), and USP7 forms a protein complex with guanosine 5′-monophosphate synthetase to catalyze the removal of H2BK120 ubiquitination (22–24). To understand the functional interplays between USP7 and the PRC2 complex in transcriptional repression in cancer cells, A375 cells stably depleted USP7 or EZH2 were infected with lentiviruses carrying FLAG-Vector or FLAG-EZH2. qChIP experiments in these cells showed that depletion of USP7 or EZH2 resulted in a reduction of the recruitment of the corresponding proteins on the promoter of co-target genes, as expected (Figure 5A). However, remarkably, knockdown of USP7 also led to a diminished recruitment of EZH2 on the promoter of the target genes (Figure 5A). Importantly, even when FLAG-EZH2 was overexpressed in USP7-deficient cells, the level of USP7 or EZH2 on the promoter of co-target genes remained to be low (Figure 5A). Meanwhile, depletion of EZH2 did not affect the recruitment of USP7 (Figure 5A). Consistently, in USP7-deficient cells, the level of H3K27me3 on the promoter of the target genes was reduced, whereas the level of H2BK120ub1 was increased (Figure 5B). Both of them could not be recovered by overexpression of EZH2 (Figure 5B). EZH2 deficiency was associated with a decrease in H3K27me3 level on the promoter of target genes, whereas the level of H2BK120ub1 on these promoters was nearly unaffected (Figure 5B).

As it is believed that USP7 is associated with PRC1, including canonical-PRC1 and non-canonical-PRC1 (such as non-canonical BCOR-PRC1.1 Complex) (34,57,58), at the same time, our silver stain results of USP7 containing RING1A also confirmed this statement (Figure 1B). To test whether the impairment recruitment of PRC2 complex induced by USP7 ablation is a secondary effect of defective H2AK119ub1, siRNA targeting USP7 were transfected into A375 cells. Western blotting showed that knockdown of USP7 had little effect on the protein level of RYBP, KDM2B, YAF2, SKP1 and PCGF1 (Figure 5C). However, depletion of USP7 resulted in a marked decrease in RING1A, RING1B and BCOR protein level (Figure 5C). Moreover, loss-of-function of RING1A, RING1B and BCOR led to the level of H2AK119ub decreased, but not H3K27me3 (Figure 5D). In addition, consistent with our earlier observations, knockdown of either RING1A, RING1B or BCOR led to a significant reduction of the binding of H2AK119ub to the target promoters, whereas any of them resulted in a limited reduction in the levels of H3K27me3 at the promoters of targets (Figure 5E).

Since RNF20 and RNF40 form a tight heterodimer that is the major E3 ligase responsible for histone H2BK120 monoubiquitination (59), in light of our observation that USP7-promoted H2BK120 deubiquitination is required for EZH2 recruitment thus H3K27 trimethylation, it is logical to postulate that RNF20/40 could influence the function of EZH2. Meanwhile, it had been reported that the EZH2 expression is controlled by the CDK9-RNF20/RNF40-H2BK120ub1 axis in a cell cycle-independent manner (60). In order to eliminate the effect on the deceased EZH2 expression following RNF20/40 deletion and to further substantiate the relationship between H3K27me3 and H2BK120ub1, A375 cells were transfected with FLAG-RNF20 or FLAG-RNF40. We found that, compared with control cells, RNF20/40 overexpressed cells displayed a weakened occupancy of H3K27me3 on the promoters of target genes, no matter the protein level of EZH2 increased or not detected by western blotting (Figure 5F and 5G). Collectively, these observations support the argument that USP7 interacts functionally with EZH2 and enables its recruitment by removal of the H2BK120ub1 mark.

Then, to explore whether USP7 deubiquitinated H2BK120ub1 for H3K27 trimethylation independent on the protein level of EZH2, USP7 knockout (*USP7*-KO) A375 cell line that we generated using CRISPER-Cas9 technology were co-transfected with FLAG-EZH2 and USP7/WT, or catalytically inactive mutant of USP7 (USP7/C223S) in a dose-dependent manner. Western blotting analysis revealed that USP7/WT, but not the catalytically inactive mutant of USP7 (USP7/C223S), could deubiquitinate H2BK120ub1 and restore the H3K27me3 level (Supplementary Figure S3A). ChIP experiments further revealed that compared to USP7/WT cells, the recruitment of EZH2 and H3K27me3 to their co-target promoters was greatly reduced and the recruitment of H2BK120ub1 was elevated in USP7/C223S cells (Supplementary Figure S3B). Overall, these results demonstrated that USP7 deubiquitinated H2BK120ub1, followed by recruiting the PRC2 complex to target promoters, supporting that USP7-regulated H2BK120ub1 deubiquitination and the physical interaction between USP7 and EZH2 are important for H3K27me3.

### Transcriptional repression of Co-targets by the USP7/PRC2 complex

**Figure 6.**
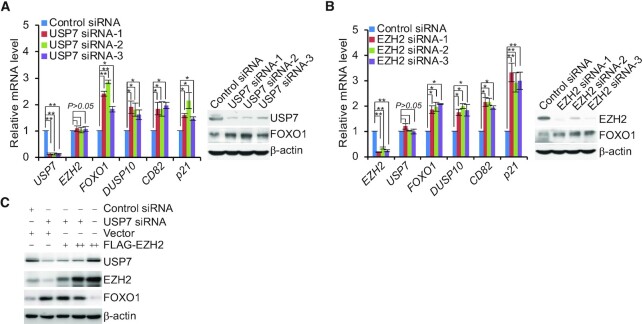
USP7 is associated with EZH2 to co-suppress the transcription of targets. (**A**) A375 cells transfected with Control siRNA or different sets of USP7 siRNAs were collected and analyzed by quantitative reverse transcription (qRT)-PCR and western blotting, respectively. (**B**) A375 cells transfected with Control siRNA or different sets of EZH2 siRNAs were collected and analyzed by qRT-PCR and western blotting, respectively. (**C**) A375 cells were co-transfected with Control siRNA or USP7 siRNA and Vector or different amounts of FLAG-EZH2; cellular extracts were collected and analyzed by western blotting. In (A and B), data represent the mean ± SD from biological triplicate experiments. *P < 0.05, **P < 0.01, one-way ANOVA.

In order to further explore the functional interaction between USP7 and the PRC2 complex to regulate transcriptional repression of co-targets, qRT-PCR and western blotting analysis revealed that knockdown of USP7 or EZH2 in A375 cells significantly increased the mRNA levels of *FOXO1, DUSP10, CD82* and *p21* (Figure 6A and B), as well as the protein level of FOXO1, as a representative target (Figure 6A and B). Moreover, the increase of FOXO1 in USP7-depleted A375 cells could not be completely rescued by overexpression of EZH2, whereas EZH2 overexpressed in cells transfected with Control siRNA significantly reduced the expression of FOXO1 (Figure 6C), suggesting that USP7-catalyzed H2BK120ub1 deubiquitination is a prerequisite for PRC2-mediated H3K27me3.

### The USP7/EZH2-FOXO1 axis is implicated in cell proliferation and tumorigenesis

**Figure 7.**
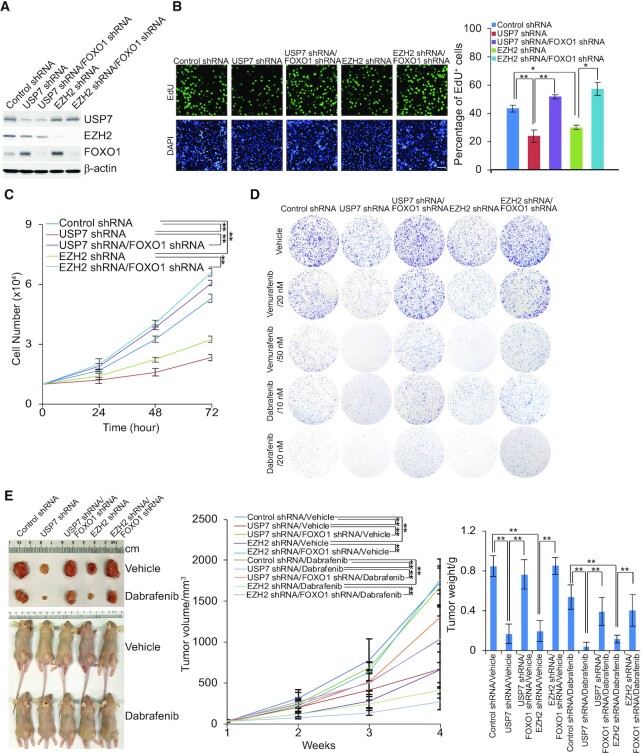
The USP7/EZH2-FOXO1 signaling pathway is required for cell proliferation and tumorigenesis. (**A**) A375 cells were infected with lentiviruses carrying the indicated shRNAs; the efficiency of knockdown was verified by western blotting. (**B**) EdU assays performed in A375 cells infected with lentiviruses carrying shRNA against the indicated targets. Representative images and statistical analysis are shown; scale bar: 50 μm. Each bar represents the mean ± SD from biological triplicate experiments. *P < 0.05, **P < 0.01, one-way ANOVA. (**C**) Growth curve assays in A375 cells were infected with lentiviruses carrying the indicated shRNAs. Each bar represents the mean ± SD from biological triplicate experiments. ** *P* < 0.01, two-way ANOVA. (**D**) A375 cells infected with lentiviruses carrying the indicated shRNAs were treated with different doses of dabrafenib or vemurafenib. Colony formation assays were performed, and the representative images are shown. (**E**) The above-described A375 cells were transplanted into athymic mice (*n* = 10); half of the mice in each group were randomly subjected to feed vehicle or dabrafenib daily one week post tumor injection. Representative tumors and sacrificed mice are shown (left panel). Tumor volumes were measured weekly and tumors were harvested and weighed when the mice were sacrificed. Error bars represent mean ± SEM, **P < 0.01, two-way ANOVA for tumor volume analysis and one-way ANOVA for tumor weight analysis (right panel).

To further explore the biological function of the USP7/PRC2 complex in cancer development and progression *in vivo*, A375 cells were infected with lentiviruses carrying Control shRNA, USP7 shRNA, EZH2 shRNA, USP7 shRNA and FOXO1 shRNA, or EZH2 shRNA and FOXO1 shRNA. The efficiency of knockdown in these cells was verified by western blotting (Figure 7A). In addition, the EdU cell proliferation assay revealed that depletion of USP7 or EZH2 in A375 cells was associated with a marked decrease in proliferating cells, whereas the effect of USP7 or EZH2 depletion on cell proliferation was partially offset when FOXO1 was knocked down (Figure 7B). Meanwhile, similar results were also obtained when A375 cells were infected with lentiviruses carrying Control shRNA, USP7 shRNA, EZH2 shRNA, USP7 shRNA and p21 shRNA, or EZH2 shRNA and p21 shRNA (Supplementary Figure S4A and S4B). Moreover, growth curve measurement indicated that the growth delay induced by USP7 or EZH2 deficiency could be partially ameliorated by FOXO1 knockdown (Figure 7C). Colony formation assays showed that USP7 or EZH2 depletion hampered the colony formation of melanoma A375 cells, which was also rescued, to some extent, by knockdown of FOXO1 (Figure 7D). Treatment of these cells with different doses of the chemotherapy drugs (BRAF inhibitor), dabrafenib or vemurafenib (61), used in melanoma treatment revealed that USP7- or EZH2-deficient cells were more sensitive to chemotherapeutics, which could be partially rescued by FOXO1 knockdown (Figure 7D).

To investigate the role of USP7 regulated-PRC2 complex in the development of melanoma and tumor sensitivity to BRAF inhibitor *in vivo*, tumors developed from A375 cell lines infected with lentiviruses carrying Control shRNA, USP7 shRNA, EZH2 shRNA, USP7 shRNA and FOXO1 shRNA, or EZH2 shRNA and FOXO1 shRNA were ectopically transplanted into 6–8 weeks old athymic mice (BABL/c nude; Charles River Laboratories) (*n* = 10). One week after inoculation, the mice were subjected to a feed vehicle or dabrafenib daily for half of the mice in each group. Then, we monitored tumor growth and mouse weights over the next 3 weeks. The results showed that, compared with control, USP7 or EZH2 knockdown was associated with a significant decrease in the growth of the primary A375 tumors, and the effect of USP7 or EZH2 depletion could be alleviated by FOXO1 knockdown (Figure 7E). Moreover, USP7 or EZH2 depletion resulted in increased sensitivity of the tumors to dabrafenib, the effect of which was largely alleviated by FOXO1 depletion (Figure 7E). Thus, USP7 recruiting PRC2 complex to suppress the expression of FOXO1 can protect melanin tumors from chemical toxicity, thus promoting the progression of melanoma.

### USP7 is implicated in tumorigenesis and poor survival of cancer patients

**Figure 8.**
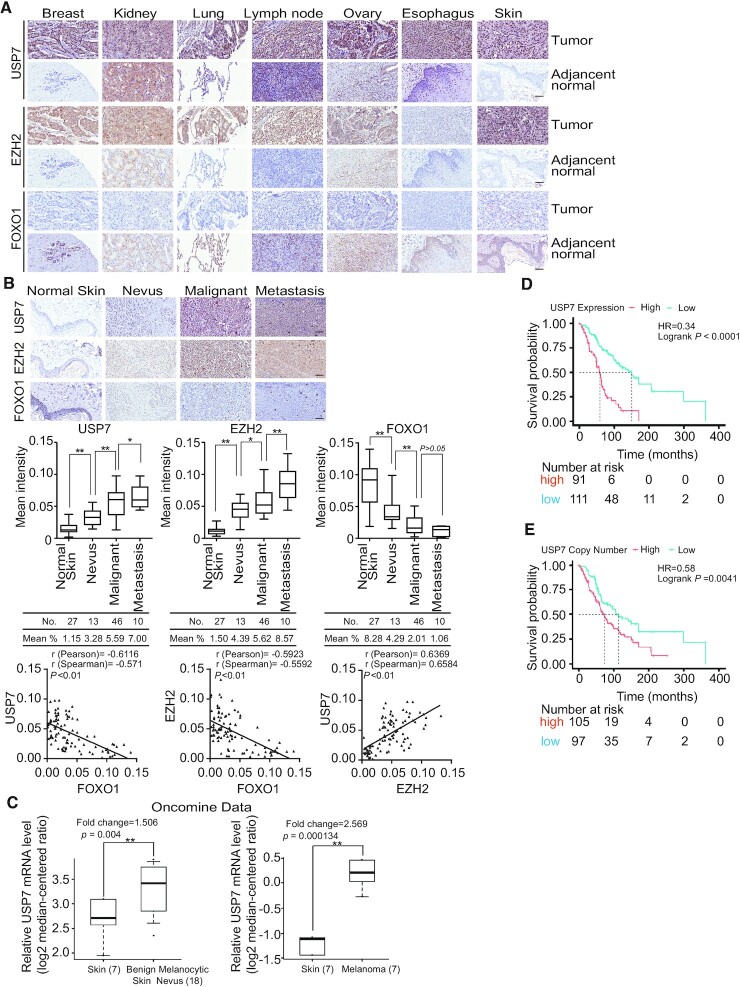
USP7 is implicated in tumorigenesis and poor patient survival. (**A**) Expression profiles of USP7, EZH2 and FOXO1 in human tissue arrays, including series of paired normal and tumor samples. Representative images (200 × magnification) from three paired samples in each case are shown; scale bar, 50 μm. (**B**) Human tissues containing normal skin, benign nevus, malignant melanoma and metastatic melanoma samples were analyzed by immunohistochemical staining. Representative images (200 × magnification) are shown; scale bar: 50 μm. The staining intensity was determined by Image-Pro Plus software and presented as box plots. *P < 0.05; **P < 0.01; one-way ANOVA. The correlation coefficient and *P* values were analyzed. (**C**) Analysis of Talantov melanoma (left panel) or Haqq melanoma (right panel) from Oncomine for the expression of *USP7* in normal human skin tissues and benign melanocytic skin nevus samples or in normal human skin tissues and melanoma samples. Data are presented as box plots (**P < 0.01). (**D**) Kaplan–Meier survival analysis for the relationship between survival time of melanoma patients and the mRNA expression level of *USP7* with survival packages from https://tcga-data.nci.nih.gov/docs/publications/tcga/. (**E**) Kaplan–Meier survival analysis of the relationship between survival time of melanoma patients and gene copy number status of *USP7* with survival packages from https://tcga-data.nci.nih.gov/docs/publications/tcga/.

FOXO1 as a tumor suppressor has been associated with multiple types of malignancies, including colon, cervical, prostate, gastric and breast cancers (44,62–65). Based on our findings that USP7 deubiquitinated H2BK120ub1 and recruited the PRC2 complex to suppress the transcription of FOXO1, we postulated that the tumorigenic effect of USP7/EZH2-FOXO1 signaling pathway could be extended to a broader scope of cancers. To validate this hypothesis, we first analyzed the protein levels of USP7, EZH2 and FOXO1 in human tissue arrays, including a series of paired tumor and adjacent normal tissue samples from the breast, kidney, lung, lymph node, ovary, esophagus and skin. Immunohistochemical staining (IHC) analysis demonstrated the clear upregulation of USP7 and EZH2 accompanied by the downregulation of FOXO1 in these cancer tissues compared to the normal tissues (Figure 8A). Next, we analyzed the expression profiles of USP7, EZH2 and FOXO1 in human samples from histologically normal skin tissues in tumor-adjacent regions, nevus, malignant melanoma and metastatic melanoma. The IHC staining and the quantitation analysis revealed that compared to the normal skin tissue, USP7 and EZH2 were highly expressed but FOXO1 was expressed at a lower level in melanoma samples, and the expression levels of USP7 with EZH2 or FOXO1 were correlated with cancer progression (Figure 8B). Moreover, analysis of the integrated cancer microarray database Oncomine (66) indicated that *USP7* mRNA levels are strikingly increased in benign melanocytic skin nevus and melanoma samples (Figure 8C). Importantly, Kaplan–Meier survival analysis from The Cancer Genome Atlas (TCGA) datasets showed that either a high expression level or increased copy number of *USP7* correlated with poor survival in melanoma patients (Figure 8D and E). Together, these findings are consistent with a role for USP7 in promoting melanoma.

## DISCUSSION

Dynamic regulation of epigenetic modifiers plays a key role in the modulation of gene expression and consequently fate specification (67,68). In particular, increased activity of the histone methyltransferase EZH2 has been associated with various cancers, including melanoma (6). In this study, we showed that EZH2 is physically associated with and is stabilized by the deubiquitinase USP7. Importantly, USP7 functions as an alternate histone modification enzyme responsible for H2BK120ub1 deubiquitination (22–24), recruiting the PRC2 complex to the chromatin, providing a molecular basis for the interplay of H2BK120ub1 and H3K27me3 in chromatin remodeling. To date, PRC2 has been shown to mediate transcriptional repression by distinct sequence-specific transcription factors (69). Interestingly, we found that FOXK1 could also physically interact with USP7 and the PRC2 complex in A375 melanoma cells (data not shown), suggesting that USP7 and PRC2 may share similar transcription factors and thus respond to the same signal pathways. However, further investigations are needed to explore the scope and variety of the functionality of the USP7/PRC2 complex and to determine whether this functionality involves additional elements.

The elevated expression of EZH2 often correlates with a poor prognosis in cancer patients (70,71). However, the mechanism of EZH2 overexpression in cancers has remained elusive. Indeed, the regulators of EZH2 expression are also critical factors for tumorigenesis. For example, Myc binds to the *EZH2* promoter and directly activates its transcription, and ANCCA, a co-activator of androgen receptor, can enhance E2F-mediated EZH2 transcription (72). Accumulating evidence indicates that the activity and stability of EZH2 are also regulated by post-translational modifications such as through CDK1/2, which could phosphorylate EZH2 at multiple sites (73), and JAK2, which phosphorylates EZH2 at tyrosine 641 to promote the interaction of EZH2 with β-TrCP and the consequent degradation of EZH2 (74). However, the protein that guarantees the stability of EZH2 has not been identified until now. USP7 has been reported to stabilize a number of proteins, thereby playing a role in multiple cellular processes (51,75). Given our finding that USP7 is physically associated with the PRC2 complex, it is reasonable that USP7 would stabilize EZH2 through its deubiquitinase activity. Future investigations will determine whether and how the USP7/PRC2 complex functions in normal development.

A classical, hierarchical model of PRC recruitment documents that initial PRC2 binding and methylation of H3K27 directs the recruitment of the ‘canonical’ PRC1 complexes, containing a CBX subunit, thereby ubiquitinating H2AK119 through the RING proteins, which in turn facilitates chromatin compaction and stable repression of non-transcribed genes (1,76–78). Reciprocally, H2AK119ub1, in several cases, is reported to modulate binding and/or catalytic activity of PRC2 through interaction with JARID2 (79), whereas loss of H2AK119ub1, in both mouse colon and skin cells, do not result in a reduction in H3K27me3 levels (58,80), pointing to an as-of-yet incompletely understood reciprocal relationship between PRC1 and PRC2.

Interestingly, like many other USP members, USP7 has been reported to deubiquitinate H2BK120ub1. Recent studies have shown that H2BK120ub1 is a prerequisite for H3K4me3 (81). Moreover, in embryonic stem cells, bivalent chromatin domains containing H3K4me3 and H3K27me3 marks silence developmental genes, while keeping them poised for activation following differentiation (82). Recently, some cancer types have been shown to exhibit partial recapitulation of bivalent chromatin modifications (83), but the roles of deubiquitinated histone H2B in histone cross-talk are poorly understood. Our findings suggest that USP7 provides a molecular basis for the interplay of H2BK120ub1 and H3K27me3 in chromatin remodeling, which is independent of the protein level of EZH2 or the level of PRC1 complex and illustrates that USP7 is likely engaged into both PRC1 and PRC2 complexes, but in a distinct subset of chromatin context or in a cell type specific manner. However, the ChIP-seq results clearly showed incomplete overlap between USP7 and EZH2 targets, suggesting that the PRC2 complex could recruit some other transcriptional cofactors. USP7 could also deubiquitinate non-histone proteins with independent functions under certain physiological states.

The FOXO1 transcription factor orchestrates the regulation of genes involved in the apoptotic response (84), cell cycle checkpoints (85) and cellular metabolism (86). FOXO1 is a putative tumor suppressor, and its gene expression is dysregulated in some cancers, including endometrial cancer (42) and melanoma (87). Consistent with our observation that USP7 coordinated with the PRC2 complex is linked to downregulation of FOXO1, we showed that the expression level of FOXO1 is decreased and negatively associated with those of USP7 and EZH2 in various tumor tissue samples. Moreover, we demonstrated that FOXO1 loss of function could partially rescue EZH2 or USP7 depletion-induced phenotypes *in vitro* and *in vivo*. Together, these findings provide a molecular basis for understanding the dysregulation of FOXO1 in carcinogenesis.

High EZH2 expression was shown to be associated with more malignant forms of tumors; thus, patients with EZH2 high expression showed significantly shorter overall survival as compared with those with lower EZH2 expression. Meanwhile, it has been reported that small-molecule inhibitors of the EZH2 histone methyltransferase are more effective in hematologic tumors than in solid tumors. In fact, cancers harboring mutations in the SNF5 subunit of the SWI/SNF chromatin remodeling complex have been shown to be available to that are currently in clinical development (88). Besides that, it has been investigated that in solid tumor, consistent reduced EZH2 activity can also promote tumorigenesis, because of significantly enhanced proliferation, DNA damage repair and activation of part of the pluripotency network, resulting in altered tumor cell identity and tumor progression, leading to concerns about the use of EZH2 inhibitors (89). Thus, a simple EZH2 inhibitor is not a suitable alternative for solid tumor, such as the BRAF inhibitor resistance melanoma treatment. On the other hand, in recent decades, patients with difficult-to-treat cancers such as advanced stage metastatic melanoma are being offered a glimpse of hope in the form of immunotherapies. By targeting factors that foster the development and maintenance of an immunosuppressive microenvironment within tumors, these therapies release the brakes on the host’s own immune system to cure the disease. Indeed, it has revealed that therapies such as ipilimumab and pembrolizumab which target the CTLA4 and PD-1 immune checkpoints, respectively, have raised the 3 years survival of patients with melanoma to 70%, and overall survival (>5 years) to 30% in phase III clinical trials (90). Despite this unprecedented efficacy, after a while, many patients fail to respond, and more concerning, some of patients who demonstrate encouraging initial responses to immunotherapy, can acquire resistance over time. There is now an urgent need to identify the molecular mechanisms of resistance, to predict outcome and to identify new targets for combination therapy. Since both USP7 and EZH2 were significantly overexpressed in a subset of patients with solid tumor, and the levels of these two factors were positively correlated, our results suggest that targeting USP7 may provide an effective treatment for patients with melanoma under a biotherapy strategy, furthermore the small-molecule inhibitors of the USP7-EZH2 protein–protein interactions to be developed may be function as potential targets for cancer therapy in the future.

In summary, we have demonstrated that USP7 is physically associated with the PRC2 complex, promoting EZH2 stabilization and chromatin engagement of the PRC2 complex to ultimately drive the development of tumors. Our findings support a model by which upregulation of USP7 in cancers resulted in an elevated abundance of EZH2, as well as H2BK120ub1 removal and an increased level of H3K27me3, which is independent on the PRC1 complex. Although it’s hard to specify whether USP7 is in dependent of or in concert with stabilizing EZH2 in PRC2 recruitment, our data indicate that USP7 coordinates with the PRC2 complex to promote tumorigenesis, supporting the pursuit of USP7 as a potential target for solid tumor intervention.

## DATA AVAILABILITY

Kaplan–Meier survival analysis of the relationship between survival time of melanoma patients and gene copy number status of *USP7* with survival packages from https://tcga-data.nci.nih.gov/docs/publications/tcga/.

All data needed to evaluate the conclusions in the paper are present in the paper and/or the Supplementary Materials. For the ChIP-seq data, they can be found at the Gene Expression Omnibus database under accession number GSE133834. Additional data related to this paper may be requested from the authors.

## Supplementary Material

gkab209_Supplemental_Files
