# Supplementary material for: Bimodal regulation of the PRC2 complex by USP7 underlies tumorigenesis
Source: Nucleic Acids Res. 2021 Apr 13;49(8):4421–40. doi: 10.1093/nar/gkab209 (PMC8096222; doi:10.1093/nar/gkab209)
Supplement: gkab209_Supplemental_Files [file gkab209_supplemental_files.zip › Supplemental Figures and Figure Legends.pdf]

Supplemental Figure 1

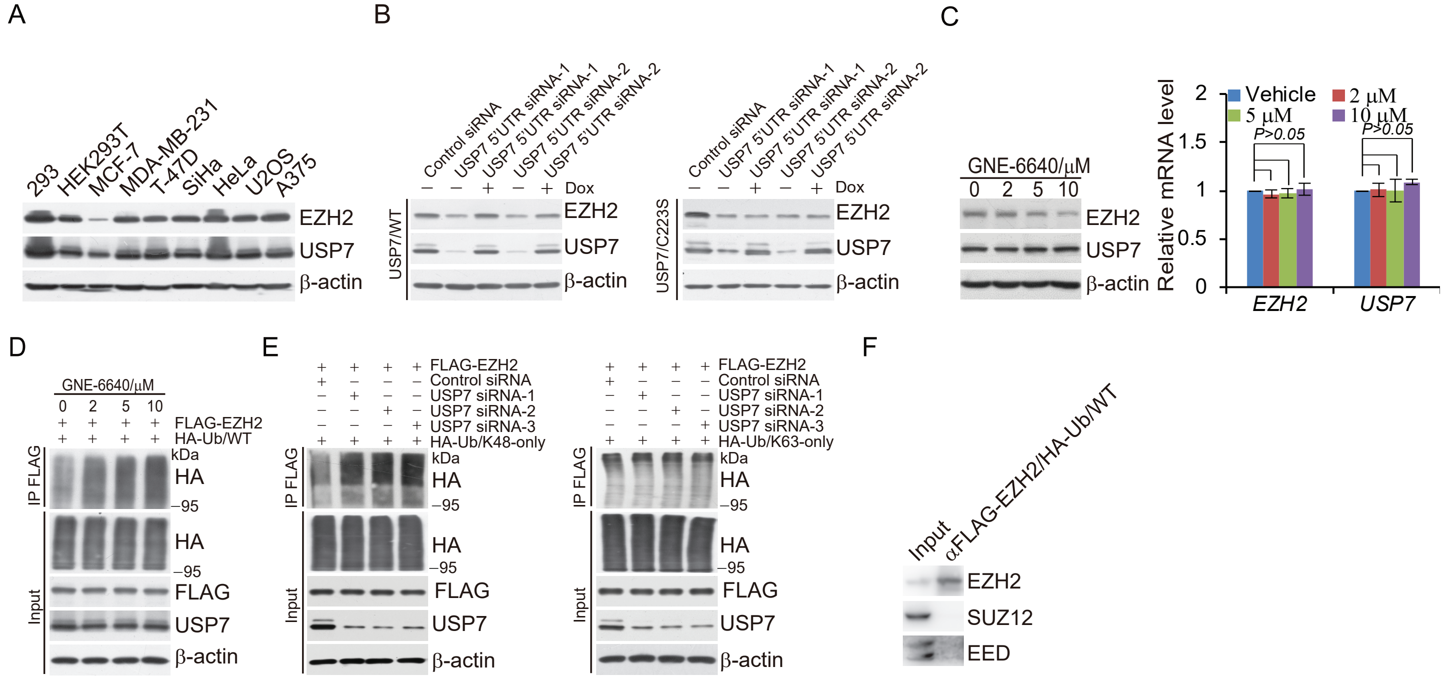

Supplemental Figure 2

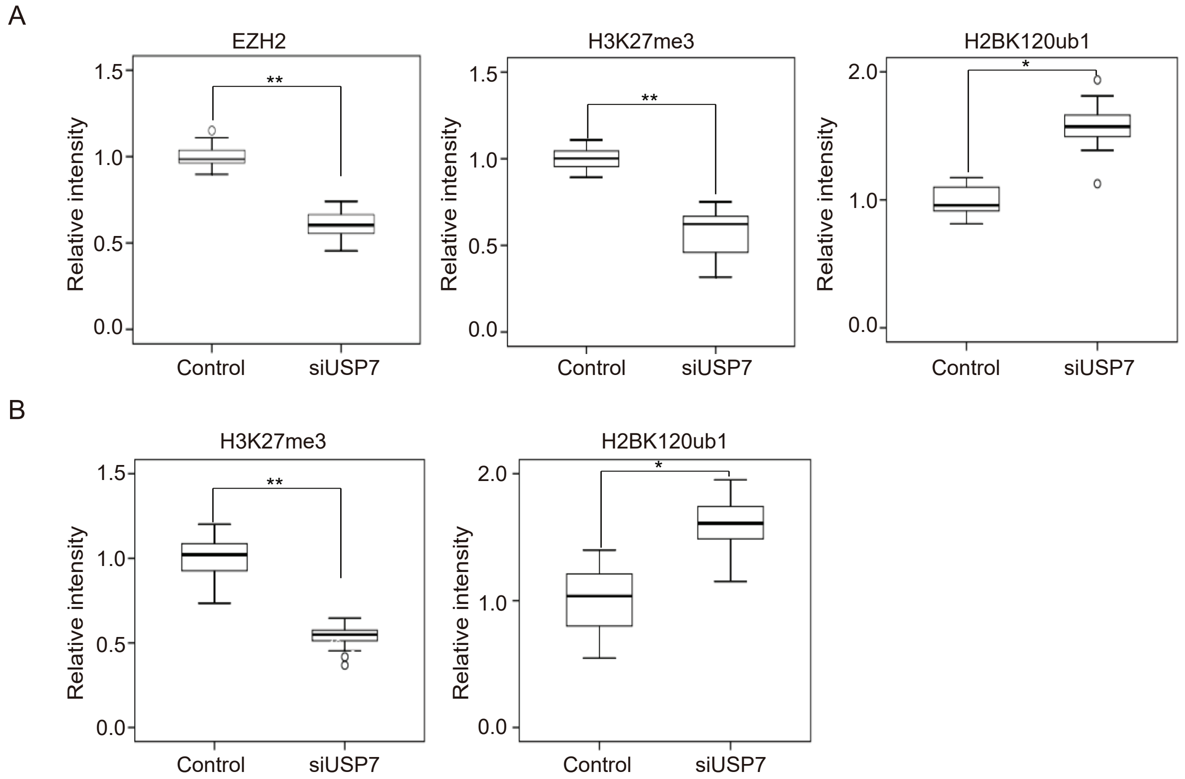

Supplemental Figure 3

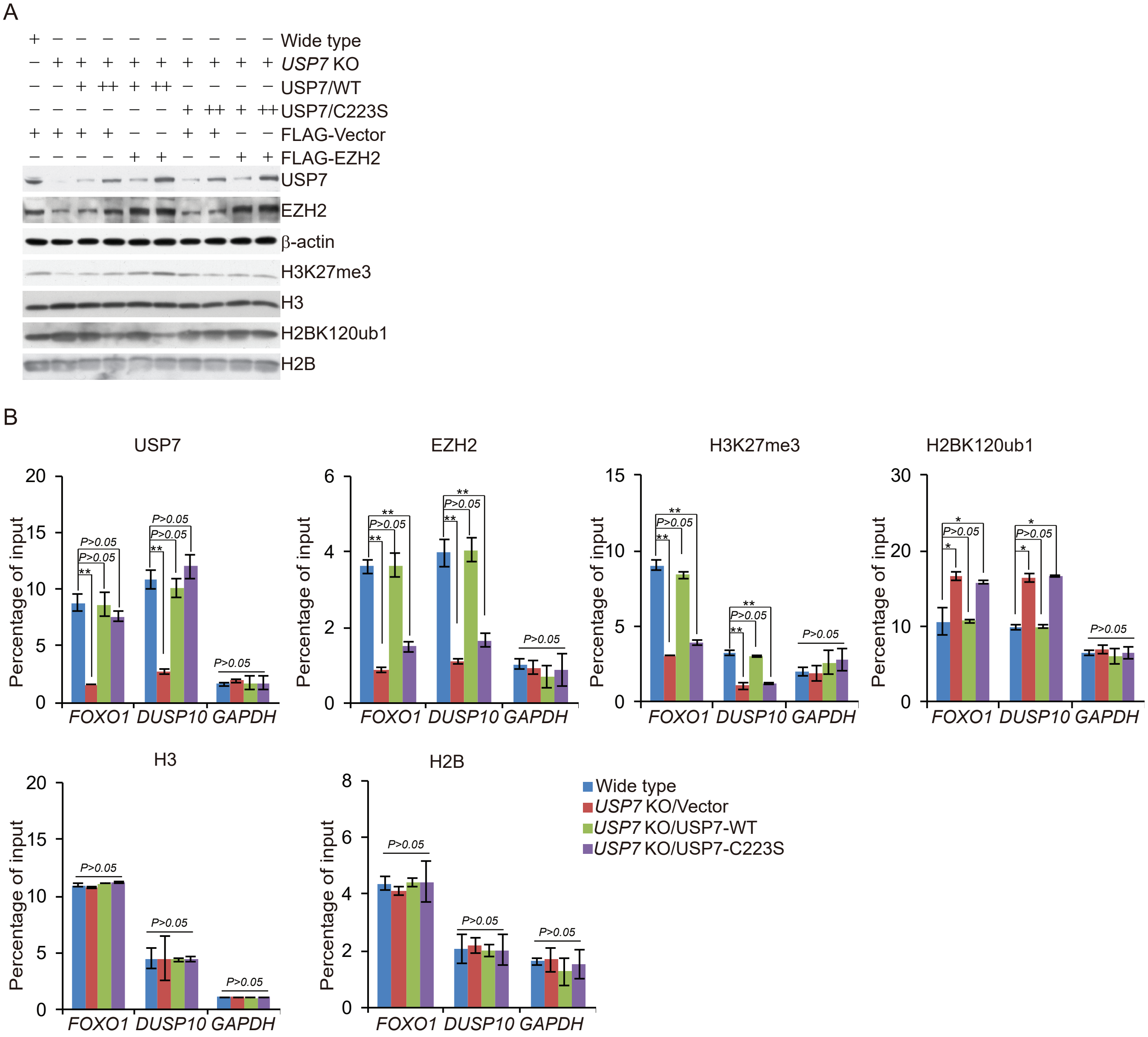

Supplemental Figure 4

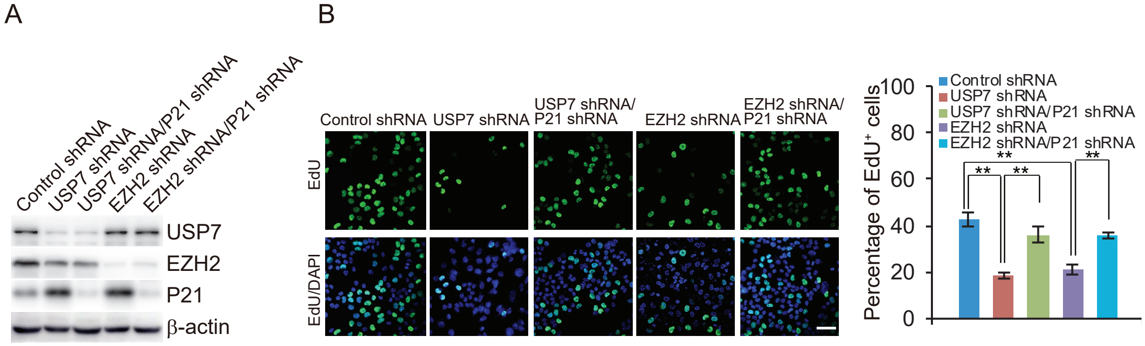

Supplemental Figure 1, related to Figure 2. USP7 promotes deubiquitination and stabilization of EZH2.

(A) Western blotting analysis of the expression of USP7 and EZH2 in multiple cell lines. (B) A375 cells with Dox-inducible expression of USP7/WT or USP7/C223S transfected with Control siRNA or different sets of USP7 5′UTR siRNAs in the absence or presence of Dox. Cellular extracts were collected and analyzed by western blotting. (C) A375 cells cultured in the absence or presence of increasing amounts of GNE-6640 for 24 hours as indicated. Cellular extracts and total RNAs were collected for western blotting and qRT-PCR analysis, respectively. Each bar represents the mean ± SD from biological triplicate experiments. *P* values were calculated by one-way ANOVA. (D) A375 cells stably expressing FLAG-EZH2 transfected with HA-Ub/WT and cultured in the presence or absence of GNE-6640. Cellular extracts immunoprecipitated with anti-FLAG followed by immunoblotting with anti-HA. (E) A375 cells stably expressing FLAG-EZH2 co-transfected with different amounts of Myc-USP7/WT and HA-Ub/K48-only or HA-Ub/K63-only followed by immunoprecipitation analysis. (F) HA-Ub-conjugated FLAG-EZH2 purified from A375 cells using high-salt and detergent buffer, the proteins were analyzed by western blotting.

Supplemental Figure 2, related to Figure 3. USP7-catalyzed H2BK120ub1 removal is a prerequisite for chromatin loading of PRC2 thus H3K27 trimethylation.

(A and B) Relative fluorescence intensities were determined by ImageJ software. 20 nuclei were scored in biological triplicate experiments. Each bar represents the mean ±

SD from biological triplicate experiments. $^{*}P < 0.05$ and $^{**}P < 0.01$, one-way ANOVA.

Supplemental Figure 3, related to Figure 5. The assembly of the USP7/EZH2 complex on transcriptional targets.

(A) Histone extracts and cellular lysates from wild type (WT), *USP7* knockout (KO) A375 cells and *USP7* KO A375 cells co-transfected with FLAG-EZH2 and different amounts of USP7/WT, or catalytically inactive mutant of USP7 (USP7/C223S) were analyzed by western blotting. Cellular and histone extracts were prepared and analyzed by western blotting. (B) qChIP analysis of selected promoters was performed using the indicated antibodies in A375 cells (WT), *USP7* knockout A375 cells transfected with Vector (*USP7* KO/Vector), USP7/WT (*USP7* KO/USP7-WT) or catalytically inactive mutant of USP7 (*USP7* KO/USP7-C223S). Each bar represents the mean ± SD from biological triplicate experiments. $^{*}P < 0.05$ and $^{**}P < 0.01$, one-way ANOVA.

Supplemental Figure 4, related to Figure 7. The USP7/EZH2-FOXO1 signaling pathway is required for cell proliferation and tumorigenesis.

(A) A375 cells were infected with lentiviruses carrying the indicated shRNAs; the efficiency of knockdown was verified by western blotting. (B) EdU assays performed in A375 cells infected with lentiviruses carrying shRNA against the indicated targets. Representative images and statistical analysis are shown. Scale bar, 50 μm. Each bar represents the mean ± SD from biological triplicate experiments. $**P < 0.01$, one-way ANOVA.
