# Supplementary material for: Bimodal regulation of the PRC2 complex by USP7 underlies tumorigenesis
Source: Nucleic Acids Res. 2021 Apr 13;49(8):4421–40. doi: 10.1093/nar/gkab209 (PMC8096222; doi:10.1093/nar/gkab209)
Supplement: gkab209_Supplemental_Files [file gkab209_supplemental_files.zip › Supplementary_Materials_.pdf]

Supplemental Information

Bimodal regulation of the PRC2 complex by USP7 underlies tumorigenesis

Dongxue Su, Wenjuan Wang, Yongqiang Hou, Liyong Wang, Xianfu Yi, Cheng Cao, Yuejiao Wang, Huan Gao, Yue Wang, Chao Yang, Beibei Liu, Xing Chen, Xiaodi Wu, Jiajing Wu, Dong Yan, Shuqi Wei, Lulu Han, Shumeng Liu, Qian Wang, Lei Shi, and Lin Shan

Contents:

Supplemental Table 1. Mass Spectrometry Analysis of USP7- or EZH2-containing Protein Complex;

Supplemental Table 2. ChIP-seq Analysis;

Supplemental Table 3. siRNA sequences;

Supplemental Table 4. Lentiviral shRNA sequences；

Supplemental Table 5. qRT-PCR primers;

Supplemental Table 6. qChIP primers

Supplementary File 1

Mass Spectrometry Analysis of USP7- or EZH2-containing Protein Complex

Mass Spectrometry Analysis of USP7-containing Protein Complex

Description	MW [kDa]	Peptide Sequence
**USP7**	128.2203389	KNIFESFVDYVAVEQLDGDNKYDAGEHGLQEAEK
		TDPKDPANYILHAVLVHSGDNHGGHYVVYLNPK
		DPANYILHAVLVHSGDNHGGHYVVYLNPK
		cTKEEAIEHNYGGHDDDLSVR
		ERQEAHLYmQVQIVAEDQFcGHQGNDMYDEEK
		HQYINEDEYEVNLKDFEPQPGNMSHPRPWLGLDHFNK
		nIFESFVDYVAVEQLDGDNKYDAGEHGLQEAEK
		DPANYILHAVLVHSGDNHGGHYVVYLNPKGDGK
		LSEVLQAVTDHDIPQQLVER
		ALDELmDGDIIVFQKDDPENDNSELPTAK
		ERQEAHLYMQVQIVAEDQFcGHQGNDMYDEEK
		NIFESFVDYVAVEQLDGDNKYDAGEHGLQEAEK
		kNIFESFVDYVAVEQLDGDNKYDAGEHGLQEAEK
		IIGVHQEDELLEcLSPATSR
		lSEVLQAVTDHDIPQQLVER
		cTKEEAIEHNYGGHDDDLSVR
		aLDELMDGDIIVFQKDDPENDNSELPTAK
		NSSLAEFVQSLSQTMGFPQDQIR
		AGFIQDTSLILYEEVKPNLTER
		iIGVHQEDELLEcLSPATSR
		ERQEAHLYmQVQIVAEDQFcGHQGNDMYDEEKVK
		IEEIPLDQVDIDKENEMLVTVAHFHK
		kNIFESFVDYVAVEQLDGDNK
		aGFIQDTSLILYEEVKPNLTER
		NQGATcYMNSLLQTLFFTNQLR
		KNIFESFVDYVAVEQLDGDNK
		SFGWETLDSFMQHDVQELcR
		sFGWETLDSFMQHDVQELcR
		GFIDDDKVTFEVFVQADAPHGVAWDSK
		eEAIEHNYGGHDDDLSVR
		QEAHLYMQVQIVAEDQFcGHQGNDMYDEEK
		ITQNPVINGNVALSDGHNTAEEDMEDDTSWR
		IEEIPLDQVDIDKENEmLVTVAHFHK
		INDRFEFPEQLPLDEFLQK
		FDKDHDVMLFLK
		hQYINEDEYEVNLK
		ERQEAHLYMQVQIVAEDQFcGHQGNDMYDEEKVK
		TMIELSDNENPWTIFLETVDPELAASGATLPK
		EEAIEHNYGGHDDDLSVR
		SLNYcGHIYTPIScK
		vFYELQHSDKPVGTKK
		AGEQQLSEPEDMEMEAGDTDDPPR
		TFRIEEIPLDQVDIDKENEMLVTVAHFHK
		nQGATcYMNSLLQTLFFTNQLR
		NSSLAEFVQSLSQTmGFPQDQIR
		HQYINEDEYEVNLK
		nSSLAEFVQSLSQTMGFPQDQIR
		KAVYMMPTEGDDSSK
		NQGATcYmNSLLQTLFFTNQLR
		SVGFFLQcNAESDSTSWScHAQAVLK
		VFYELQHSDKPVGTKK

		vFYELQHSDKPVGTK
		kAVYMMPTEGDDSSK
		tFRIEEIPLDQVDIDKENEMLVTVAHFHK
		RPAMLDNEADGNK
		TRSLNYcGHIYTPIScK
		iQSLLDIQEKEFEK
		NIFESFVDYVAVEQLDGDNK
		RPAmLDNEADGNK
		ALDELMDGDIIVFQKDDPENDNSELPTAK
		GVKFLTLPPVLHLQLMR
		SFGWETLDSFmQHDVQELcR
		VFYELQHSDKPVGTK
		SDRREDYYDIQLSIK
		rIQSLLDIQEKEFEK
		FDKDHDVmLFLK
		IQSLLDIQEKEFEK
		EEEITLYPDKHGcVR
		hcTNAYMLVYIR
		nQGATcYmNSLLQTLFFTNQLR
		fDKDHDVMLFLK
		nSSLAEFVQSLSQTmGFPQDQIR
		RIQSLLDIQEKEFEK
		iEEIPLDQVDIDKENEMLVTVAHFHK
		KAVYmmPTEGDDSSK
		YDAGEHGLQEAEK
		HTGYVGLKNQGATcYMNSLLQTLFFTNQLR
		sLNYcGHIYTPIScK
		iNDRFEFPEQLPLDEFLQK
		rEDYYDIQLSIK
		KAVYMmPTEGDDSSK
		NQGATcYMNSLLQTLFFTNQLRK
		QEAHLYMQVQIVAEDQFcGHQGNDMYDEEKVK
		IQSLLDIQEKEFEKFK
		HcTNAYmLVYIR
		iRDLLPVMcDR
		HcTNAYMLVYIR
		ENDWGFSNFMAWSEVTDPEK
		AGEQQLSEPEDMEmEAGDTDDPPR
		DFEPQPGNMSHPRPWLGLDHFNK
		rIQSLLDIQEK
		FLTLPPVLHLQLMR
		lNTDPMLLQFFK
		SLNYcGHIYTPIScKIRDLLPVMcDR
		nIFESFVDYVAVEQLDGDNK
		LNTDPMLLQFFKSQGYR
		LSESVLSPPcFVR
		aLDELmDGDIIVFQK
		cIWLNSQFREEEITLYPDK
		RIQSLLDIQEK
		REDYYDIQLSIK
		SLNYcGHIYTPIScKIR
		RISHLFFHK
		ESKLSEVLQAVTDHDIPQQLVER
		VLKNSSLAEFVQSLSQTMGFPQDQIR
		IRDLLPVMcDR

		iEEIPLDQVDIDKENEmLVTVAHFHK
		LNTDPMLLQFFK
		TIPNDPGFVVTLSNR
		rISHLFFHK
		MNYFQVAKTVAQR
		kAVYmMPTEGDDSSK
		DHDVMLFLK
		SEATFQFTVER
		FmYDPQTDQNIK
		IRDLLPVmcDR
		FEFPEQLPLDEFLQK
		DFEPQPGNmSHPRPWLGLDHFNK
		ALDELmDGDIIVFQK
		yDAGEHGLQEAEK
		sEATFQFTVER
		EVFGTFGIPFLLR
		eNDWGFSNFMAWSEVTDPEK
		TIPNDPGFVVTLSNRMNYFQVAK
		DLYHRVDVIFcDK
		aLDELmDGDIIVFQKDDPENDNSELPTAK
		AGEQQLSEPEDmEmEAGDTDDPPR
		lSESVLSPPcFVR
		aLDELMDGDIIVFQK
		ALDELMDGDIIVFQK
		AVYMMPTEGDDSSK
		fLTLPPVLHLQLMR
		sDRREDYYDIQLSIK
		eVFGTFGIPFLLR
		DLLEEcKKAVELGEK
		SQGYRDGPGNPLR
		IQSLLDIQEK
		DHDVmLFLK
		iQSLLDIQEK
		mKGTcVEGTIPK
		aVYMMPTEGDDSSK
		EDYYDIQLSIK
		WcKFDDDVVSR
		fEFPEQLPLDEFLQK
		ISHLFFHK
		hTGYVGLK
		eEEITLYPDK
		IINYRDDEK
		AVYmmPTEGDDSSK
		tIPNDPGFVVTLSNR
		VLLDNVENKMK
		MVSYIQcKEVDYR
		FLTLPPVLHLQLmR
		kLYYQQLK
		HNYEGTLRDLLQFFKPR
		GKmVSYIQcK
		GTcVEGTIPKLFR
		LNTDPmLLQFFK
		eDYYDIQLSIK
		mKGTcVEGTIPK
		fmYDPQTDQNIK

		DDPENDNSELPTAK
		FDDDVVSR
		iQDYDVSLDK
		mNYFQVAK
		FDKDHDVMLFLKMYDPK
		AVYmMPTEGDDSSK
		dLLQFFKPR
		DLLQFFKPR
		cIWLNSQFR
		DGPGNPLRHNYEGTLR
		IQDYDVSLDK
		AVELGEKASGKLR
		fDDDVVSR
		HTGYVGLK
		TmIELSDNENPWTIFLETVDPELAASGATLPK
		EEEITLYPDK
		ITQNPVINGNVALSDGHNTAEEDmEDDTSWR
		IEEIPLDQVDIDK
		FMYDPQTDQNIK
		aVYmMPTEGDDSSK
		VLLDNVENK
		fDKDHDVmLFLK
		DLLPVMcDR
		lNTDPmLLQFFK
		fAIVMMGR
		VDVIFcDK
		iSHLFFHK
		fYPDRPHQK
		YTYLEKAIK
		LLEIVSYK
		MNYFQVAK
		vLLDNVENK
		mNYFQVAK
		lLEIVSYK
		iRDLLPVmcDR
		kAVYmmPTEGDDSSK
		GTcVEGTIPK
		HNYEGTLR
		KLYYQQLK
		sFGWETLDSFmQHDVQELcR
		iINYRDDEK
		dFEPQPGNMSHPRPWLGLDHFNK
		FAIVMmGR
		mVSYIQcK
		MKITDFENR
		fMYDPQTDQNIK
		vDVIFcDK
		dDPENDNSELPTAK
		MVSYIQcK
		FAIVmmGRHQYINEDEYEVNLK
		hNYEGTLR
		fLTLPPVLHLQLmR
		dLLPVMcDR
		dGPGNPLR
		FAIVmmGR

		FAIVMMGR
		FDDDVVSRcTK
		hcTNAYmLVYIR
		DLLPVmcDR
		DGPGNPLR
		YTVFKVLK
		KLYYQQLKMK
		ITDFENR
		fAIVmMGR
		FYPDRPHQK
		mYDPKTRSLNYcGHIYTPIScK
		mVSYIQcK
		YTYLEKAIKIHN
		LYYQQLK
		iHQGEHFR
		rPAmLDNEADGNK
		fAIVmmGR
		lYYQQLK
		VLKNSSLAEFVQSLSQTmGFPQDQIR
		lNTDPmLLQFFKSQGYR
		IHQGEHFR
		FKFAIVmMGR
		iTDFENRR
		iTDFENR
		IHQGEHFREVmKR
		gTcVEGTIPK
		VKYTVFK
		dHDVMLFLK
		sRYTYLEK
		DLLEEcKK
		ITDFENRR
		iMVMPR
		IHQGEHFREVmK
		LWPMQAR
		LWPmQAR
		dLLEEcKK
		gKmVSYIQcK
		mKITDFENR
		SVPLALQR
		mKITDFENRR
		ImVMPR
		YTYLEK
		yTYLEK
		IMVMPR
		ImVmPR
		imVMPR
		lWPMQAR
		imVmPR
		DLLEEcK
		sVPLALQR
		KAVELGEK
**SUZ12**	80.2712023	NSESLHQENKPGSVKPTQTIAVK
		HGGGGGGGSGPSAGSGGGGFGGSAAVAAATASGGK
		ALETDSVSGVSK
		STAPIAKPLATR

		TITQIEEFSDVNEGEKEVMK
		QPGFAFSR
		ESLTTDLQTR
		FIFNYVYHPK
		LYSLLK
**EZH2**	80.98643099	IQPVHILTSVSSLR
		FANHSVNPNcYAK
		EFAAALTAER
**CBX4**	61.3294698	RREEEVSGVSDPQPQDAGSR
		GYLGAVKPLAGAAGAPGK
		LLIAFQNR
**RING1A**	39.12115473	SLRPDPNFDALISK
		FcSDcIVTALR
**EED**	45.49530777	VTLYEcHSQGEIR
		IKPSESNVTILGR
		EVSTAPAGTDMPAAK
		DPNLLLSVSK
		IMScGMDHSLK
		MLALGNQVGK

Mass Spectrometry Analysis of EZH2-containing Protein Complex

Description	MW [kDa]	Peptide Sequence
**FLNA**	279.8	AEFTVETR
		AEGPGLSR
		AEISFEDR
		AEISFEDRK
		AFGPGLQGGSAGSPAR
		AGNNMLLVGVHGPR
		AGVAPLQVK
		ALTQTGGPHVK
		ANLPQSFQVDTSK
		AYGPGIEPTGNMVK
		DAGEGGLSLAIEGPSK
		DAPQDFHPDR
		DVDIIDHHDNTYTVK
		EAGAGGLAIAVEGPSK
		EATTEFSVDAR
		ENGVYLIDVK
		ETGEHLVHVK
		FADQHVPGSPFSVK
		FVPAEMGTHTVSVK
		GAGTGGLGLAVEGPSEAK
		GEITGEVR
		GEYTLVVK
		GKLDVQFSGLTK
		IANLQTDLSDGLR
		IQQNTFTR
		LSPFMADIR
		LTVSSLQESGLK
		LVSIDSK
		NDNDTFTVK
		NGHVGISFVPK
		SPFEVYVDK
		TGVAVNKPAEFTVDAK

		VANPSGNLTETYVQDR
		VAQPTITDNKDGTVTVR
		VDVGKDQEFTVK
		VEPGLGADNSVVR
		VHGPGIQSGTTNKPNK
		VNQPASFAVSLNGAK
		VTAQGPGLEPSGNIANK
		VTVLFAGQHIAK
		VYGPGVAK
		YAPSEAGLHEMDIR
		YGGQPVPNFPSK
		YNEQHVPGSPFTAR
		AWGPGLEGGVVGK
		SPFEVK
		TGVELGKPTHF
		VAQPTITDNK
		YTILIK
		VKETADFK
		AIVDGNLK
		SQQLAPQYTY
		FTIDTK
		SVAVSPSLDLSK
		YGGPYHIGGSPFK
		GPGLEGGVVGK
		LDVQFSGLTK
		DKGEYTLVVK
		YGGDEIPFSPYR
		VTVLFAGQH
		LQTDLSDGLR
		TGVELGKPTHFTVNAK
		FTVETR
		DQEFTVK
		FADQHVPGSPF
		AGGPGLER
		TVGPLGEGGAHK
		LYSVSYLLK
		RLTVSSLQESGLK
		AGDQPSVQPPLR
		GDEHIPGSPYR
		VTAQGPGLEPSGN
		APGSYLISIK
		SVTIDGPSK
		VNVGAGSHPNK
		AGQSAAGAAPGGGVDTR
		FADQATPTSPIR
		LLGWIQNK
		VKAEGPGLSR
		LTETYVQDR
		LKPGAPLRPK
		ANLPQSF
		GIEPTGNMVK
		DLAEDAPWK
		AHEPTYF
		KGEITGEVR
		YNEQHVPGSPF

		VVQEPGDYEVSVK
		SLAIEGPSK
		TVAYVPDVTGR
		VGEPGHGGDPGLVSAY
		SLSIEGPSK
		SLNVTYGGH
		GPGLQGGSAGSPAR
		ADQATPTSPIR
		TGVAVNKPAEF
		TGVELGKPTHFTVN
		VSGLGEK
		YTAPQPGK
		ALAGDQPSVQPPLR
		DLSDGLR
		LSQFPK
		DGTVTVR
		FVPAEMGTH
		SQQLAPQYTYAQ
		VGISFVPK
		AEFTVETR
		AEGPGLSR
		AEISFEDR
		AEISFEDRK
		AFGPGLQGGSAGSPAR
		AGDQPSVQPPLR
		AGGPGLER
		AGNNMLLVGVHGPR
		AGQSAAGAAPGGGVDTR
		AGVAPLQVK
		AIVDGNLK
		ALTQTGGPHVK
		ANLPQSFQVDTSK
		AYGPGIEPTGNMVK
		DAPQDFHPDR
		DGTVTVR
		DKGEYTLVVK
		DLAEDAPWK
		DQEFTVK
		EAGAGGLAIAVEGPSK
		EATTEFSVDAR
		ENGVYLIDVK
		FTIDTK
		FTVETR
		FVPAEMGTHTVSVK
		GAGTGGLGLAVEGPSEAK
		GPGVAKTGLK
		IQQNTFTR
		KGEITGEVR
		LLGWIQNK
		LSPFMADIR
		LTVSSLQESGLK
		LVSIDSK
		NDNDTFTVK
		NGHVGISFVPK
		SPFEVK

		SPFEVYVDK
		SVTIDGPSK
		TGVAVNKPAEFTVDAK
		TVGPLGEGGAHK
		VAQPTITDNK
		VDVGKDQEFTVK
		VEPGLGADNSVVR
		VHGPGIQSGTTNKPNK
		VNQPASFAVSLNGAK
		VNVGAGSHPNK
		VTAQGPGLEPSGNIANK
		VTVLFAGQHIAK
		VYGPGVAK
		YGGQPVPNFPSK
		YNEQHVPGSPFTAR
		YTILIK
**SPTBN1**	274.4	AFEDEMSGR
		AKDEQSAVSMLK
		ALVADSHPESER
		DGMAFNALIHK
		DLVAIEAK
		DVSSVELLMNNHQGIK
		EIGQSVDEVEK
		FESLEPEMNNQASR
		GEQVSQNGLPAEQGSPR
		HEVSASTQSTPASSR
		HRPDLIDFDK
		HYASEEIK
		IDDIFER
		ITDLYTDLR
		KQALQDTLALYK
		LEDLEVIQHR
		LEMNLGLQK
		LESEHPDQAQAILSR
		LLEVLSGER
		LLTQHENIK
		LNDGNEYLFQAK
		LQAAYAGDKADDIQK
		LVDTGDKFR
		NEIDNYEEDYQK
		QIEAQEKPR
		QLQEDAAR
		SGHFEQAIK
		TALPAQSAATLPAR
		TQILAASYELHK
		TQTAIASEDMPNTLTEAEK
		VAVVNQIAR
		VLDNAIETEK
		VLVLSQDYGK
		YKEVAELTR
		DTGNIGQER
		HQAFMAELASNK
		LISDINK
		LQQFLR
		QALQDTLALYK

		DLTSVNILLK
		LVSDGNINSDR
		LPEELGR
		ALQFLK
		ETASELLMR
		LLQLTEK
		MLTAQDMSYDEAR
		ELEAENYHDIK
		FSALER
		KQQMLENQMEVR
		VHLENMGSHDIVDGNHR
		FMELLEPLNER
		VQAVVAVAR
		LFQLNR
		INAVVETGR
		DQNTVETLQR
		HSEVPVSLK
		DEQSAVSMLK
		EGEDMIAEEHFGSEK
		MAETVDTSEMVNGATEQR
		LQAAYAGDK
		KLPEELGR
		EGMQLISEKPETEAVVK
		SAATWDER
		LVDTGDK
		QQMLENQMEVR
		ENEVLEAWK
		ILSSDDYGK
		LLELLR
		RLVSDGNINSDR
		NQTLQK
		VDSIDDR
		LILEVHQF
		QLMHSGHPSEK
		KQEDFMTTMDANEEK
		LSGIEER
		AYQQFLR
		RPPSPEPSTK
		DLTSVMR
		LFDANK
		ETWLSENQR
		QNLLSQSH
		LYAGLK
		ALAVEGK
		ANLEQLSAIR
		VADSHPESER
		EASLGEASK
		FSLFGK
		LEQLAR
		EVLESTTQTK
		INAVVETGRR
		AFEDEMSGR
		DLVAIEAK
		AKDEQSAVSMLK
		ALAVEGK

		ALQFLK
		ALVADSHPESER
		AYQQFLR
		DGMAFNALIHK
		DQNTVETLQR
		DLTSVMR
		DTGNIGQER
		EGEDMIAEEHFGSEK
		EIGQSVDEVEK
		ELEAENYHDIK
		ETASELLMR
		ETWLSENQR
		FESLEPEMNNQASR
		FSALER
		HEVSASTQSTPASSR
		HRPDLIDFDK
		IDDIFER
		ILSSDDYGK
		INAVVETGR
		KLPEELGR
		KQEDFMTTMDANEEK
		LAEISDVWEEMK
		LEMNLGLQK
		LEQLAR
		LFDANK
		LFQLNR
		LISDINK
		LLELLR
		LLEVLSGER
		LLQLTEK
		LLTQHENIK
		LNDGNEYLFQAK
		LPEELGR
		LQAAYAGDK
		LQQFLR
		LSGIEER
		LVDTGDKFR
		LVSDGNINSDR
		MAETVDTSEMVNGATEQR
		MLTAQDMSYDEAR
		QIEAQEKPR
		QLQEDAAR
		QQMLENQMEVR
		SGHFEQAIK
		TALPAQSAATLPAR
		VAVVNQIAR
		VDSIDDR
		VLDNAIETEK
		VLVLSQDYGK
		VQAVVAVAR
**USP7**	128.2	DDPENDNSELPTAK
		RPAMLDNEADGNK
		VLLDNVENK
		YTYLEK
		FDDDVVSR

		LLEIVSYK
		DGPGNPLR
**EZH2**	81	EFAAALTAER
		QVYEFR
		YSQADALK
		PSTPTINVLESK
		FANHSVNPN
		EFAAALTAER
		EFAAALTAER
		EFAAALTAER
		YSQADALK
**OGT**	106.8	AATGEEVPR
		AFLDSLPDVK
		DSGNIPEAIASYR
		EQGNIEEAVR
		IIFSPVAPK
		ISSPLFNTK
		IVLNGIDLK
		LVSIVADQLEK
		NRQEYEDIAVK
		SAHFSTLAIK
		SDLGNLLK
		TIIVTTR
		ASSVGNVADSTEPTKR
		ASSVGNVADSTEPTK
		REQGNIEEAVR
		EYQAGDFEAAER
		SNGHIYDNR
		QYTMELER
		QEYEDIAVK
		AVAAYLR
		LGTDLEYLK
		QGLAELAHR
		NELFALR
		ALSPDDGTNFR
		VTMPGETLASR
		IKPVEVTESA
		AATGEEVPR
		ASSVGNVADSTEPTKR
		AVAAYLR
		EQGNIEEAVR
		IIFSPVAPK
		ISSPLFNTK
		IVLNGIDLK
		LASVLQQQGK
		LGTDLEYLK
		LGTDLEYLKK
		LVSIVADQLEK
		NELFALR
		QEYEDIAVK
		QYTMELER
		SAHFSTLAIK
		SDLGNLLK
		TIIVTTR
**SUZ12**	80.3	ALETDSVSGVSK

		ESLTTDLQTR
		FIFNYVYHPK
		QPGFAFSR
		SYSLLFR
		KLYSLLK
		NSESLHQENKPGSVKPTQTIAVK
		TFVAQMTVFDK
		LYSLLK
		QVPLNPDLNQTK
		NLIAPIFLHR
		VDDMLSK
		ATWETILDGK
		ALETDSVSGVSK
		ESLTTDLQTR
		FIFNYVYHPK
		QPGFAFSR
		TFVAQMTVFDK
**PRMT5**	72.6	AAMAVGGAGGSR
		VPLVAPEDLR
		GPLVNASLR
		SDLLLSGR
		LYNEVR
		YSQYQQAIYK
		GFPVLSK
		QPITVR
		EFIQEPAK
		GPLVNASLR
		SDLLLSGR
		VPLVAPEDLR
**EED**	45.5	DEVLSADYDLLGEK
		DPNLLLSVSK
		DPVSPNLR
		ESYDYNPNK
		EVSTAPAGTDMPAAK
		HYVGHGNAINELK
		IHFPDFSTR
		IKPSESNVTILGR
		MLALGNQVGK
		TNRPFISQK
		PLVFATVGSNR
		PSESNVTILGR
		SADYDLLGEK
		TSHPLLAVAGSR
		EGDPLVFATVGSNR
		LYVWDLEVEDPHK
		DLEVEDPHK
		QTSFSR
		MEDDIDK
		PNLLLSVSK
		FSMDFWQK
		LGDLILSK
		WLGDLILSK
		TERPDTPTNTPNAPGR
		TYDSNTSHPLLAVAGSR
		DPNLLLSVSK

		EGDPLVFATVGSNR
		ESYDYNPNK
		EVSTAPAGTDMPAAK
		FSMDFWQK
		HYVGHGNAINELK
		IHFPDFSTR
		IKPSESNVTILGR
		LLLSVSK
		LYVWDLEVEDPHK
		MEDDIDK
		MLALGNQVGK
		PSESNVTILGR
		TNRPFISQK
		TSHPLLAVAGSR
**MTF2**	65.2	AEKEPEGTSHEFK
		ALQTQNSEIVK
		EPEGTSHEFK
		IDSSVIDSDEK
		LHPGELADTPK
		NSITSYFGAAGR
		SVGRPPGPYTR
		TEGTAHSSNTSDVDFTGASSAK
		VPPVPPNVAFK
		YEHVLEALNDYK
		SSNTSDVDFTGASSAK
		HYGLSDSR
		ETTSSSISR
		HLFGLR
		AEKEPEGTSHEFK
		ALQTQNSEIVK
		LHPGELADTPK
		NSITSYFGAAGR
		VPPVPPNVAFK

Supplemental Table 3. siRNA sequences

siRNAs	Sequences
USP7-1	GACGUUUCGAAUAGAGGAA
USP7-2	GCACUAAUGCUUACAUGUU
USP7-3	GACUUUGAGAACAGGCGAA
USP7 5’UTR-1	CUCACCUCGUCAGCCACUA
USP7 5’UTR-2	CAAGUCUUGUGUUUAGGCU
EZH2-1	AGAAUACUUGAACUUGUCC
EZH2-2	GAGGUUCAGACGAGCUGAU
EZH2-3	GCAACACCCAACACUUAUAAG
RING1B-1	CCCUUAGAAGUGGCAACAA
RING1B-2	GCAGCGAGGCAAGAAACAA
RING1A-1	GCCCUGAUCUCUAAGAUCUAU
RING1A-2	CUGGAGCUGGUGAAUGAGAAA
BCOR-1	GCCAAAUAAGUAUUCACUGAA
BCOR-2	CCACGAAACUUAUACUUUCAA

Supplemental Table 4. Lentiviral shRNA sequences

shRNAs	Sequences
Control	CCGGGATATGGGCTGAATACAAACTCGAGTTTGTATTCAGCCCATATCTTTTTG
USP7	CCGGCCTGGATTTGTGGTTACGTTACTCGAGTAACGTAACCACAAATCCAGGTTTTTG
EZH2	CCGGGCAACACCCAACACTTATAAGCTCGAGCTTATAAGTGTTGGGTGTTGCTTTTTG
FOXO1	CCGGCCAAACACCAGTTTGAATTCTCGAGAATTCAAACTGGTGTTTGGTTTTTG
P21	CCGGCAGGCGGUUAUGAAAUUCACTCGAGUGAAUUUCAUAACCGCCUGTTTTTG

Note: Red color indicates the targeting sequence against the corresponding genes.

Supplemental Table 5. qRT-PCR primers

Genes	Forward Primer Sequences	Reverse Primer Sequences
USP7	ATTCCTAACATTGCCACCAG	ATTTACACCATTTGCCATCC
EZH2	GTACACGGGGATAGAGAATGTGG	GGTGGGCGGCTTTCTTTATCA
FOXO1	TCGTCATAATCTGTCCCTACACA	CGGCTTCGGCTCTTAGCAAA
DUSP10	ATCGGCTACGTCATCAACGTC	TCATCCGAGTGTGCTTCATCA
p21	CGATGGAACTTCGACTTTGTCA	GCACAAGGGTACAAGACAGTG
CD82	TGTCCTGCAAACCTCCTCCA	CCATGAGCATAGTGACTGCCC
GAPDH	GAAGGTGAAGGTCGGAGTC	GAAGATGGTGATGGGATTTC

Supplemental Table 6. qChIP primers

Genes	Forward Primer Sequences	Reverse Primer Sequences
FOXO1	CAGCCCAGATGAGGAAAGG	CGGGAGATAGGACCAAAGC
DUSP10	TTCTTTAGGGTTCTTGGACG	AAAGTTGCCCTGGGATACA
p21	AGGTTCAAGCGATTCTCC	ATCACAGGGTCAGGAGTT
CD82	CCCTGACTTCTTTACAGTTTGC	GGAACTTGTTAGGAGTGCGTAT
BAI1	CACTGTGACGGGTAAATTATGG	ACCTCTGCCCTTCCTCTGC
MOB2	GTGTCTGTGCTGGGTTGGTG	ATGGGAGTTGTGGTGTCTGG
DAB2IP	GGGCAAATACTTGAAATGG	CCTCCCTGGTGACCTTGT
DLG3	GATTTGGGTTTGCGTGTT	GGAATGGAGGAAGGATGAC
HRK	ACCCTTACCTACCTGTGCCT	CCTTGCCTCTGCTGCCTA
NISCH	GAGCTACAAAGTCGTGGGC	TTATCTGGAAAGTGGAGGTCAT
GAPDH	TCTGCTCTGGGTGGTCATTGTGAA	TGCTAAGTTTAGCCTGCCTGGTGA
